# A novel FRET peptide assay reveals efficient *Helicobacter pylori* HtrA inhibition through zinc and copper binding

**DOI:** 10.1038/s41598-020-67578-2

**Published:** 2020-06-29

**Authors:** Sabine Bernegger, Cyrill Brunner, Matej Vizovišek, Marko Fonovic, Gaetano Cuciniello, Flavia Giordano, Vesna Stanojlovic, Miroslaw Jarzab, Philip Simister, Stephan M. Feller, Gerhard Obermeyer, Gernot Posselt, Boris Turk, Chiara Cabrele, Gisbert Schneider, Silja Wessler

**Affiliations:** 1 Microbiology, Department of Biosciences, University of Salzburg, Billrothstrasse 11, 5020 Salzburg, Austria; 2 Institut für Pharmazeutische Wissenschaften, ETH Zürich, Vladimir-Prelog-Weg 4, 8093 Zurich, Switzerland; 3 Department of Biochemistry and Molecular and Structural Biology, Jozef Stefan Institute, Jamova 39, 1000 Ljubljana, Slovenia; 4 Organic Chemistry and NMR Spectroscopy for Protein Research, Department of Biosciences, University of Salzburg, Billrothstrasse 11, 5020 Salzburg, Austria; 5 Biological Systems Architecture Group, Department of Oncology, Weatherall Institute of Molecular Medicine, University of Oxford, Oxford, UK; 6 Tumor Biology Unit, Institute of Molecular Medicine, Charles Tanford Protein Center, Martin-Luther-University Halle-Wittenberg, Halle, Germany; 7 Membrane Physics, Department of Biosciences, University of Salzburg, Billrothstrasse 11, 5020 Salzburg, Austria; 8 Present Address: University of Milan, Via Festa del Perdono 7, 20122 Milan, Italy; 9 Present Address: Dipartimento Di Farmacia, Università Di Napoli “Federico II”, Via D. Montesano, 49, 80131 Naples, Italy

**Keywords:** Drug screening, Antimicrobials, Pathogens

## Abstract

*Helicobacter pylori* (*H. pylori*) secretes the chaperone and serine protease high temperature requirement A (HtrA) that cleaves gastric epithelial cell surface proteins to disrupt the epithelial integrity and barrier function. First inhibitory lead structures have demonstrated the essential role of HtrA in *H. pylori* physiology and pathogenesis. Comprehensive drug discovery techniques allowing high-throughput screening are now required to develop effective compounds. Here, we designed a novel fluorescence resonance energy transfer (FRET) peptide derived from a gel-based label-free proteomic approach (direct in-gel profiling of protease specificity) as a valuable substrate for *H. pylori* HtrA. Since serine proteases are often sensitive to metal ions, we investigated the influence of different divalent ions on the activity of HtrA. We identified Zn^++^ and Cu^++^ ions as inhibitors of *H. pylori* HtrA activity, as monitored by in vitro cleavage experiments using casein or E-cadherin as substrates and in the FRET peptide assay. Putative binding sites for Zn^++^ and Cu^++^ were then analyzed in thermal shift and microscale thermophoresis assays. The findings of this study will contribute to the development of novel metal ion-dependent protease inhibitors, which might help to fight bacterial infections.

## Introduction

Gastric cancer is associated with one of the highest mortality rates among all cancerous diseases in humans since efficient treatment options are still not available^1^. Persistent infections with the gastric pathogen *Helicobacter pylori* (*H. pylori*) are a significant risk factor for the induction and progression of stomach cancer. Approximately 50% of the human population is infected with *H. pylori*, which can induce chronic gastritis, duodenal, and gastric ulcers and finally gastric adenocarcinoma or MALT (mucosa-associated lymphoid tissue) lymphoma^2,3^. Accordingly, the complex network of cellular and molecular mechanisms of *H. pylori*–host interactions has been intensively investigated.

The finding that the serine protease high temperature requirement A (HtrA) expressed by *H. pylori* targets cell surface proteins of infected host cells added an important aspect to the model of *H. pylori* pathogenesis. During infection, *H. pylori* secretes HtrA and cleaves off the ectodomain of the cell adhesion protein and tumor suppressor E-cadherin, which was identified as the first HtrA substrate significant for pathogenesis^4^. E-cadherin is the key molecule of adherens junctions and necessary for establishing and maintaining intact intercellular adhesions between epithelial cells. Loss of E-cadherin function has drastic consequences not only on the epithelial architecture, but also on tumor prevention through the lack of recruitment of cancer-associated signal transduction molecules like β-catenin or p120-catenin^5,6^. In fact, E-cadherin ectodomain shedding leads to the disintegration of intercellular adhesion and promotes malignity of gastric, pancreatic, or breast cancer^7,8^. Therefore, E-cadherin cleavage serves as a reliable cancer biomarker^9,10^.

Structurally, E-cadherin is composed of an extracellular domain (EC), a transmembrane domain (TMD) and an intracellular domain (IC). The EC domain consists of the five tandem repeats EC1–EC5 with interspaced calcium-binding motifs, which are required for functional homophilic *cis* and *trans* interactions of E-cadherin between epithelial cells^6^. Importantly, these sites have been identified as preferred signature motifs for *H. pylori* HtrA^11^. Later studies indicated that the presence of calcium ions efficiently blocks E-cadherin cleavage by interfering with the accessibility of calcium-binding regions representing HtrA cleavage sites^12^. Additional HtrA substrates, including fibronectin, occludin, and claudin-8, have been described, confirming the capability of HtrA to break open intercellular adhesions and to disrupt the integrity of the epithelial barrier^13^. As a consequence, HtrA paves the intercellular way for *H. pylori* to transmigrate across the epithelial layer and to facilitate β1-integrin-mediated delivery of the bacterial oncoprotein cytotoxin-associated gene A (CagA)^4,13^.

HtrA proteins are widely expressed and their role in bacterial pathogenesis is well established. The HtrA proteins of several pathogens have been suggested to process adhesins, as *htrA* deletion mutants of *Streptococcus pneumoniae*, *Listeria monocytogenes*, *Staphylococcus aureus*, etc*.* show a reduced ability to colonize host cells or tissues^14,15^. Similar findings have been reported for *Shigella flexneri* DegP, which is important for the surface exposure of the virulence factor and autotransporter intra*/*intercellular spread protein A (IcsA)^16^. *Chlamydia trachomatis* secretes HtrA from chlamydial inclusions into the host cytoplasm, where it plays a critical role in the chlamydial developmental cycle^17,18^. However, HtrA-mediated E-cadherin cleavage appears to be a prevalent mechanism since similar observations have also been made for *Campylobacter jejuni*, enteropathogenic *Escherichia coli* (EPEC), *Salmonella enterica* subsp. *enterica* ser. Typhimurium, *Yersinia enterocolitica*, and *Proteus mirabilis*^19–22^.

The structure and regulation of prokaryotic HtrA proteases have already been extensively studied in *E. coli*, which expresses the three homologues DegP, DegQ, and DegS^23^. DegP proteins in Gram-negative bacteria comprise of an N-terminal signal peptide, followed by a chymotrypsin-like protease domain with the catalytic triad histidine, aspartic acid, and serine residues. Substrate recognition, binding, and HtrA/DegP homo-oligomerization are mainly mediated by the two C-terminally located PDZ (Post synaptic density of 95 kDa, Discs large, Zonula occludens-1) modules^24^. HtrA/DegP protease activity has been associated with the formation of higher order multimers composed of trimers. DegP is thought to form inactive hexameric oligomers that are converted into proteolytic active oligomers consisting of 12–24 HtrA monomers upon substrate binding^25–27^.

Computational drug design has identified functional HtrA inhibitors docking either into the active pocket of HtrA or interfering with an allosteric ligand-binding site that is important for HtrA oligomerization^4,28,29^. The development of a substrate-derived peptide inhibitor further emphasized the significance of HtrA activity in *H. pylori* pathogenesis^11^. Importantly, bacterial HtrA also functions as a chaperone that refolds and degrades misfolded proteins under stress conditions^30^. Due to this important function in bacterial physiology, HtrA expression is essential for *H. pylori*. This is obviously a unique phenotype for *H. pylori*^31,32^ as deletion of the *htrA* gene from the bacterial genome has not yet been reported to be lethal for other bacteria. The unexpected finding that small molecule inhibitors targeting HtrA can efficiently block *H. pylori* growth and survival^31^ indicates that *H. pylori* HtrA could be an attractive target for screening of pharmacological inhibitors.

In this study, we established a novel fluorescence assay based on Förster resonance energy transfer (FRET) that is suitable for high-throughput screenings and determining the effect of divalent ions on the activity of HtrA. Previous studies have reported that Zn^++^ can directly block the activity of serine proteases and potentiate moderate serine protease inhibitors by chelating the inhibitor to the histidine and serine of the catalytic triad in the active center^33^. In our study, we found that Zn^++^ and Cu^++^ can block HtrA activity and hence, we hypothesize that Zn^++^ or Cu^++^ could function as a co-inhibitor of HtrA proteases.

## Results and discussion

### A novel FRET peptide assay to determine the activity of *H. pylori* HtrA

**Figure 1:**
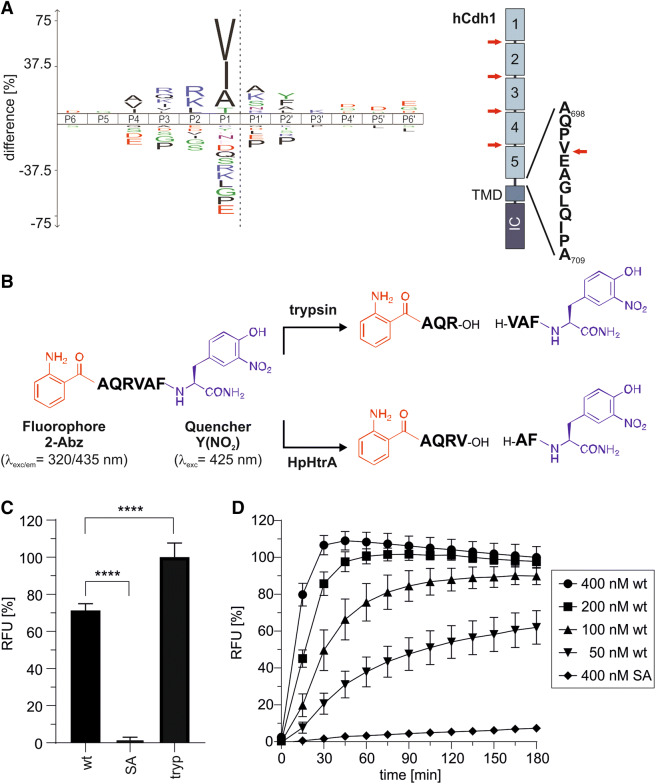
A novel FRET peptide assay to detect *H. pylori* HtrA activity. (**A**) The cleavage specificity profile of HpHtrA obtained from DIPPS profiling is represented as an iceLogo, with significantly enriched and under-represented amino acids above and below the *x*-axis, respectively. The scissile peptide bond between P1 and P1′ is shown as a gray dashed line in the iceLogo. The model on the right represents the domain structure of human E-cadherin (hCdh1), which is composed of the extracellular domain (EC1–EC5), a transmembrane domain (TMD) and an intracellular domain (IC). HtrA cleavage sites have been identified (red arrows) in the Ca^++^-binding sites located between the individual EC regions. According to the iceLogo, an additional cleavage site for HtrA is present in the linker region between the EC5 domain and TMD. (**B**) The sequence AQRVAF harboring 2-aminobenzoyl (2-Abz) as fluorophore and 3-nitro-tyrosine Y(NO_2_) as a quencher is hydrolyzed by trypsin with arginine (R) at position P1 and by HpHtrA with valine (V) at position P1. (**C**) 5 µM of the FRET peptide were incubated with 250 nM of HpHtrA wild type (wt), its isogenic inactive mutant (SA), or 125 nM trypsin for 180 min in 50 mM HEPES buffer (pH 7.4) at 37 °C. The data represent the relative fluorescent units (RFU) ± S.D. with the fluorescent signal obtained from trypsin-treated FRET peptide set as 100%. Asterisks indicate statistically significant differences (****p < 0.0001). (**D**) 4 µM of the FRET peptide were incubated with indicated concentrations of HpHtrA wt or SA for 180 min at 37 °C in 50 mM HEPES buffer (pH 7.4). The data represent the RFU ± S.D. with the fluorescent signals obtained from FRET peptide treated with 400 nM HpHtrA wt for 180 min set as 100%.

So far, the activity of *H. pylori* HtrA (HpHtrA) has been mainly investigated by casein zymography or Western blot analyses of substrate fragments, which are laborious, slow, and low-throughput methods^28,34^. FRET technology represents state-of-the-art methodology and allows continuous assays of protease activity and high-throughput screening of protease inhibitors. To develop a FRET peptide assay containing an optimized short cleavage site for HtrA, we performed global specificity profiling for HtrA using a direct in-gel profiling of protease specificity (DIPPS) assay^35^. Analyzing HtrA-targeted proteome-derived peptides from MKN-28 cells, we detected 2,479 peptides that were processed by HtrA. These peptides were commonly cleaved after the aliphatic amino acid residues V, I and A in P1 position, while preference for basic amino acid residues such as R and K was observed in P2 position. Specificity of other non-prime binding sites was less pronounced, with R showing the highest enrichment at the P3 position and A at P4. Among the prime sites, the amino acids A and K were shown to be enriched at P1′ position and Y at P2′ position (Fig. 1A, left panel, Fig. S1). The detected characteristic specificity profile AR/QRV↓AY corresponds well with the signature site [VITA]↓[VITA]-x-x-D-[DN] previously identified in the HtrA substrate E-cadherin and verifies the preference of HpHtrA to cleave between hydrophobic amino acids^11^. Interestingly, the sequence AR/QRV↓AY resembles the AQPVEA linker region between the EC5 and the TMD of E-cadherin that has already been suggested as a preferred cleavage site in E-cadherin for HpHtrA during the infection process^11,12^, but has not yet been experimentally verified (Fig. 1A, right panel). Based on this refined cleavage site pattern, a FRET peptide consisting of the optimized sequence AQRVAF, the N-terminal fluorophore 2-aminobenzoyl (2-Abz) and the C-terminal quencher 3-nitro-tyrosine Y(NO_2_) was synthesized (Fig. S2). This was equally well cleaved by trypsin and by HpHtrA with R and V, respectively, in position P1 (Fig. 1B and Fig. S3 and S4). Intramolecular fluorescence quenching of the FRET peptide was highly efficient (Fig. S3) and cleavage using trypsin and HtrA wild type (wt) revealed a strong increase in emitted fluorescence, whereas the inactive mutant of HtrA (SA) did not affect the FRET substrate (Fig. 1C). Mass-spectrometry analyses demonstrated HtrA-specific cleavage with V in the P1 position (Fig. S4). The kinetics of the proteolytic activity of increasing HpHtrA wt concentrations demonstrated effective cleavage of the FRET peptide already at low concentrations compared to the inactive HtrA SA mutant (Fig. 1D).

### The divalent cations Zn^++^ and Cu^++^ block HpHtrA-mediated E-cadherin cleavage

**Figure 2:**
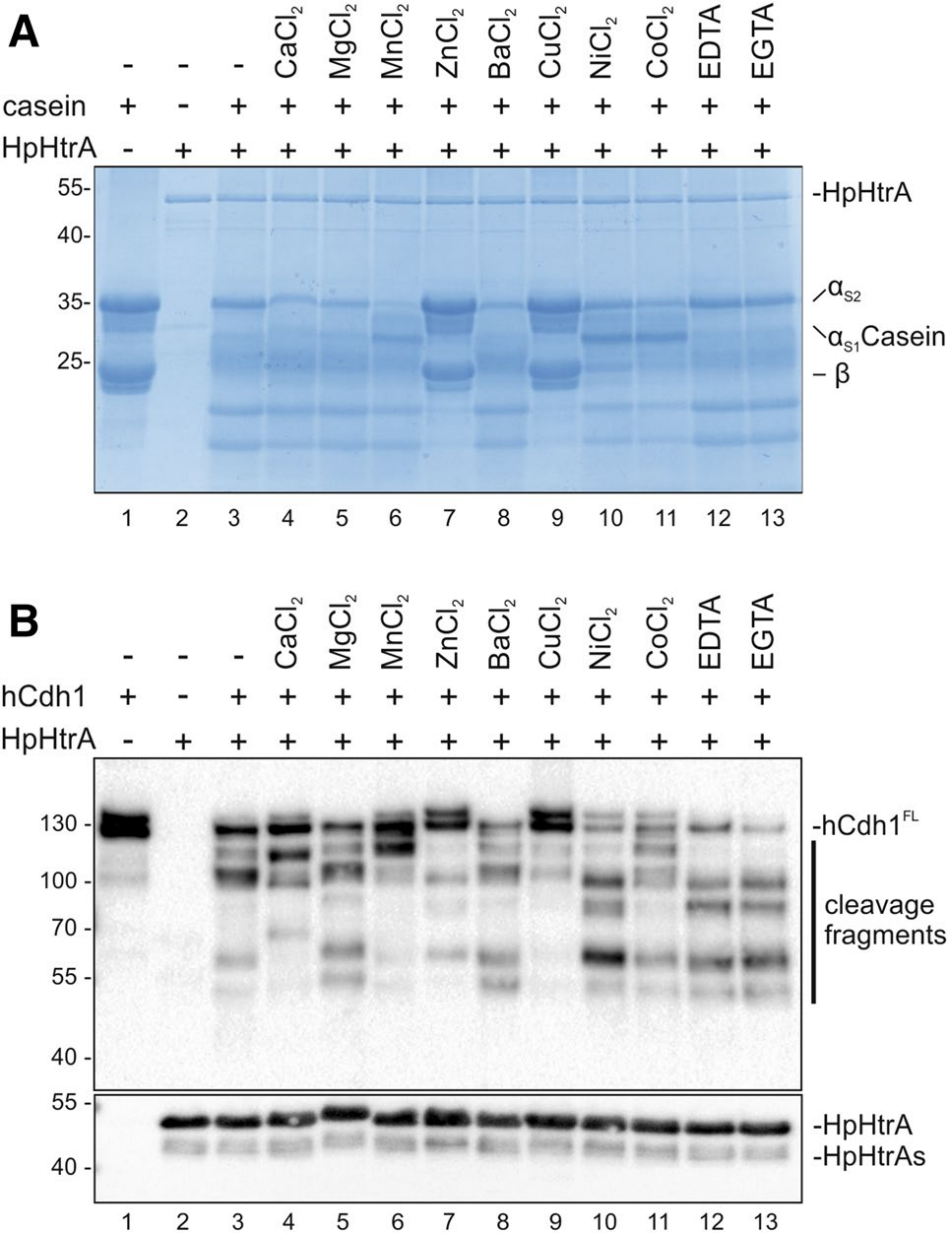
Divalent cations modulate the activity of HpHtrA. (**A**) 10 µg casein composed of α_S1_-, α_S2_- and β-casein were incubated with 250 ng HpHtrA for 16 h at 37 °C in 50 mM HEPES buffer (pH 7.4). Where indicated, 1 mM of the different divalent ions, EDTA, or EGTA was added. Proteins were separated by SDS-PAGE and stained with Coomassie Brilliant Blue G250. (**B**) 50 ng hCdh1 were incubated with 250 ng HpHtrA for 16 h at 37 °C in 50 mM HEPES buffer (pH 7.4). Where indicated, 1 mM of the different divalent ions, EDTA, or EGTA was added. Full length hCdh1 (Cdh1^FL^, 125 kDa) and hCdh1 cleavage fragments were detected by Western blot using an antibody recognizing the EC5 domain of hCdh1. HpHtrA and the auto-processed HpHtrA (HpHtrAs) were detected using a polyclonal HpHtrA antibody.

The FRET peptide assay is a suitable approach for drug screening to identify and optimize HpHtrA inhibitors. A wide range of non-metalloproteases can be inhibited by Zn^++^, including trypsin, kallikrein, and thrombin^33,36,37^. Following our observation that calcium ions efficiently prevent E-cadherin ectodomain shedding by covering the HpHtrA-targeted signature sites in the substrate^11,12^, we carried out a detailed investigation of the effects of divalent ions on *H. pylori* HtrA activity and substrate cleavage. In a first approach, recombinant HpHtrA was incubated with casein composed of α_S1_-, α_S2_-, and β-casein as a substrate and cleavage was analyzed in Coomassie-stained protein gels (Fig. 2A). HpHtrA efficiently degraded β-, α_S1_- and, to a lesser extent, α_S2_-casein (Fig. 2A, lane 3). Among the metal ions tested, Zn^++^ and Cu^++^ strongly inhibited HtrA activity, indicating a direct effect of these divalent cations on the proteolytic activity of HpHtrA (Fig. 2A, lanes 7 and 9). The chelating agents EDTA and EGTA were added as controls, and these did not alter HtrA-mediated casein degradation (Fig. 2A, lanes 12 and 13). These data imply that divalent transition metals can differentially influence the activity of HpHtrA. Casein degradation by HtrA expressed by *Borrelia burgdorferi* (BbHtrA) is similarly inhibited by Zn^++^ and Cu^++^, and has also been observed for DegP from *E. coli* and human HtrA1^38^. Interestingly, the crystal structure of the HtrA homolog A (HhoA) from the photosynthetic cyanobacterium *Synechocystis* sp. PCC 6,803 revealed that the hexameric structure contains a Zn^++^ ion within the central channel, which inactivates HhoA in a casein cleavage assay^39^. These data support our findings that HpHtrA can be inhibited through direct binding of Zn^++^ and Cu^++^.

To determine whether this inhibition is caused through direct effects on the HtrA protease or through interference with the HtrA substrate casein, we further analyzed the effect of the metal ions on HtrA-mediated E-cadherin cleavage. Recombinant human E-cadherin (hCdh1) was incubated with recombinant HpHtrA for 16 h in the presence of various divalent cations as indicated. Fragments of hCdh1 were detected by Western blot analyses using an antibody recognizing the EC5 domain of E-cadherin. Efficient HpHtrA-mediated cleavage of hCdh1 was indicated by the presence of the characteristic HtrA-induced fragmentation pattern^11^ (Fig. 2B, lane 3). The addition of Ca^++^ significantly blocked hCdh1 cleavage, while the chelating agents EDTA and EGTA considerably enhanced hCdh1 fragmentation compared to HpHtrA wt alone, as previously reported^12^ (Fig. 2B, lanes 4, 12 and 13). A drastic inhibitory effect was observed after the addition of Zn^++^ and Cu^++^ since HpHtrA-mediated hCdh1 fragmentation was strongly decreased (Fig. 2B, lanes 7 and 9). This is consistent with the in vitro cleavage experiments using casein as a substrate (Fig. 2A). These data suggest that Zn^++^ and Cu^++^ interfere with HpHtrA activity rather than bind to the substrate. This is obviously in contrast to Ca^++^, which binds to the Ca^++^-binding motifs positioned between the individual EC domains. Binding of Ca^++^ is required for functional intermolecular interactions in *cis* and *trans* between the extracellular domains of E-cadherin on adjacent epithelial cells. In particular, these Ca^++^-binding motifs have been previously identified as cleavage sites for HtrA. Hence, Ca^++^ binding masks the motifs and prevents further fragmentation of the soluble 90 kDa extracellular domain of E-cadherin upon infection with *H. pylori*^11,12^.

**Figure 3:**
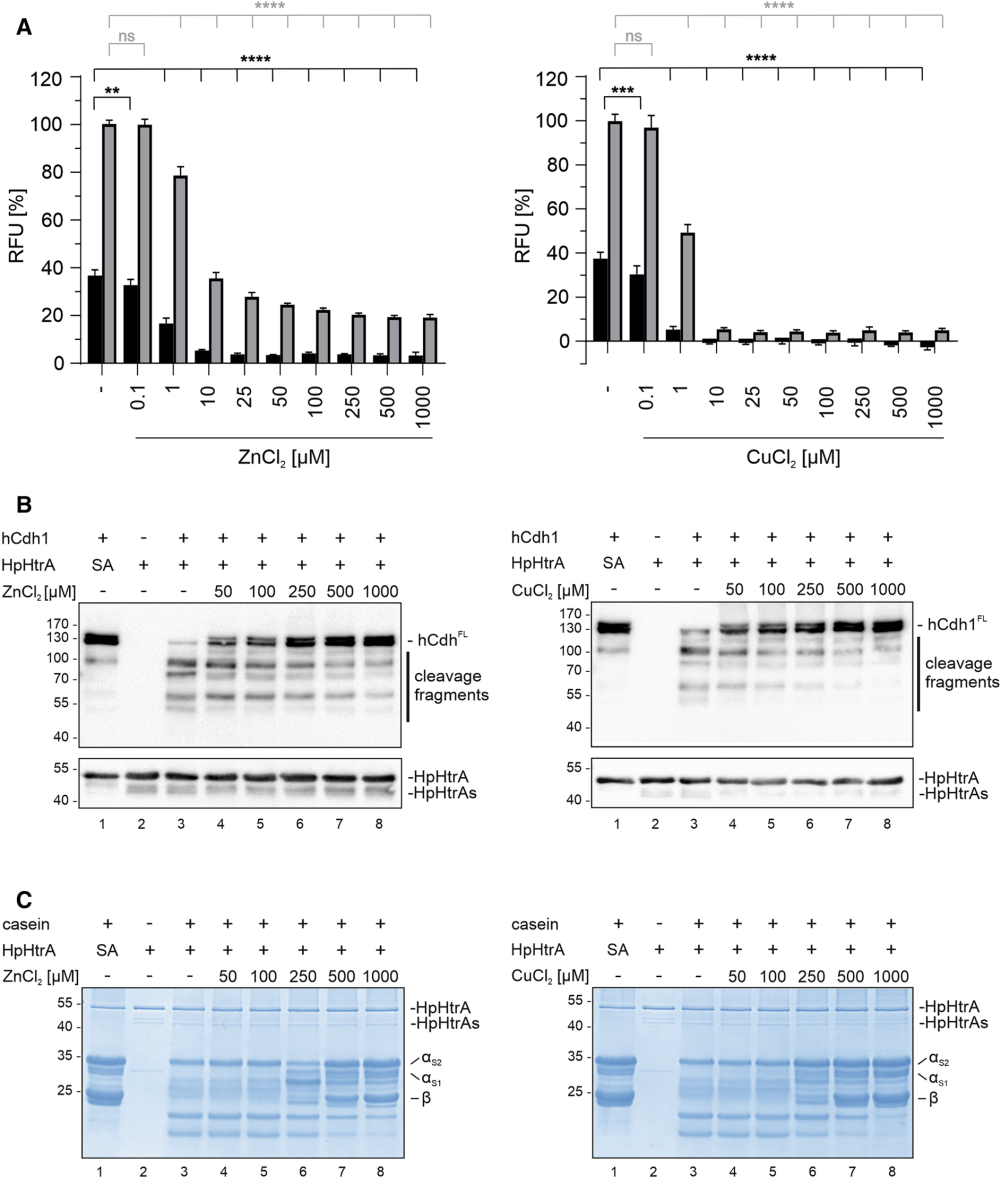
ZnCl_2_ and CuCl_2_ inhibit HpHtrA activity in a concentration-dependent manner. (**A**) 5 µM FRET peptide were incubated with 250 nM HpHtrA and increasing concentrations of ZnCl_2_ (left panel) or CuCl_2_ (right panel) for 15 min (black bars) and 180 min (grey bars) at 37 °C in 50 mM HEPES buffer (pH 7.4). The data represent the relative fluorescence units (RFU) ± S.D. with fluorescent signals obtained from FRET peptide treated with HpHtrA wt set as 100%. Asterisks indicate statistically significant differences (****p < 0.0001; ns, non-significant). (**B**) 50 ng hCdh1 were incubated with 250 ng HpHtrA wt or inactive mutant (SA) and increasing concentrations of ZnCl_2_ (left panel) or CuCl_2_ (right panel) for 16 h at 37 °C in 50 mM HEPES buffer (pH 7.4). Full length hCdh1 (Cdh1^FL^) and cleavage fragments were detected by Western blot using an antibody recognizing the EC5 domain of hCdh1. HpHtrA and the auto-processed short HpHtrA (HpHtrAs) were detected using a polyclonal antibody. (**C**) 10 µg casein composed of α_S1_-, α_S2_- and β-casein were incubated with 250 ng HpHtrA wt or inactive mutant (SA) and with increasing concentrations of ZnCl_2_ (left panel) or CuCl_2_ (right panel). After incubation at 37 °C for 16 h proteins were separated by SDS-PAGE and visualized by staining with Coomassie Brilliant Blue G250.

In order to further refine the inhibitory action of Zn^++^ and Cu^++^, we performed titration experiments to determine the concentrations of Zn^++^ and Cu^++^ that are required for HtrA inhibition using the FRET peptide assay (Fig. 3A). Importantly, addition of Zn^++^ and Cu^++^ alone did not quench the fluorescent signal (Fig. S5), confirming the specificity of the measurement and the putative broad application of the FRET peptide for future drug screening. The relative fluorescence after 15 min and 180 min demonstrated that increasing concentrations of Zn^++^ and Cu^++^ significantly hampered HtrA activity (Fig. 3A). The half maximal inhibitory concentration (IC_50_) values calculated for Zn^++^ were 649.5 nM (95% confidence interval 560–746.1 nM) after 15 min and 2.57 µM (95% confidence interval 2.382–2.774 µM) after 180 min. Cu^++^ exhibited an IC_50_ of 295.4 nM (95% confidence interval 255.8–342.7 nM) after 15 min and of 937.5 nM (95% confidence interval 890.9–980.4 nM) after 180 min, indicating efficient HpHtrA inactivation. The inhibition of HpHtrA wt detected in the FRET peptide assay was also compared with in vitro cleavage experiments using hCdh1 or casein as substrates. As a control, we included a proteolytically inactive HpHtrA mutant (SA)^4,40^, which does not process hCdh1 or casein. Zn^++^ clearly inhibited HpHtrA-mediated hCdh1 fragmentation at a concentration of 50 µM (Fig. 3B, lane 4) and higher Zn^++^ concentrations blocked hCdh1 cleavage even more efficiently (Fig. 3B, lanes 5–8). In contrast to hCdh1 cleavage, the caseinolytic activity of HpHtrA was not affected by low Zn^++^ or Cu^++^ concentrations (Fig. 3C) suggesting that casein can absorb Zn^++^ and Cu^++^ as previously reported^41^ and hence interfere with cleavage activity. A slight inhibitory effect could be detected using 250 µM Zn^++^ or Cu^++^, which was further enhanced at increasing concentrations (Fig. 3C, lanes 6–8). An increase up to 500–1,000 µM was required to fully inhibit casein cleavage (Fig. 3C, lanes 7–8). The different concentrations required for efficient HpHtrA inhibition detected in the FRET peptide assay and the in vitro cleavage experiment might be due to the increased accessibility of the FRET peptide, which contains a single, conformation-independent consensus cleavage site rather than folded complex proteins. Therefore, we conclude that using the FRET peptide as a substrate for HpHtrA is a highly sensitive and reliable approach to quantify HtrA activity.

The IC_50_ values of Zn^++^ and Cu^++^ inhibition are not within physiological concentrations (free plasma concentrations are ~ 10^−9^ mol/l for Zn^++^ and ~ 1.6 × 10^−11^ mol/l for Cu^++^)^42^. However, in combination with serine protease inhibitors, zinc has been reported to be a potent intensifier of moderate small molecule inhibitors^33,37,43^. Even though not all serine proteases contain typical Zn^++^-binding sites, which are characterized by two histidine residues with their imidazole side-chains as ligands for Zn^++^, it has been suggested that Zn^++^ and Cu^++^ bind in the active pockets of thrombin^33^, trypsin^33^, and kallikrein^36^ and bridge the inhibitor to the active site leading to an efficient inhibition. These studies demonstrated that lower concentrations of both, inhibitor and Zn^++^, were sufficient to strongly potentiate inhibitory effects. Since we found that Zn^++^ blocks HpHtrA activity, it is tempting to speculate that moderate HtrA inhibitors combined with a low concentration of Zn^++^ ions may synergistically function as highly potent zinc-mediated serine protease inhibitors.

### Zn^++^ and Cu^++^ differentially interact with HpHtrA

**Figure 4:**
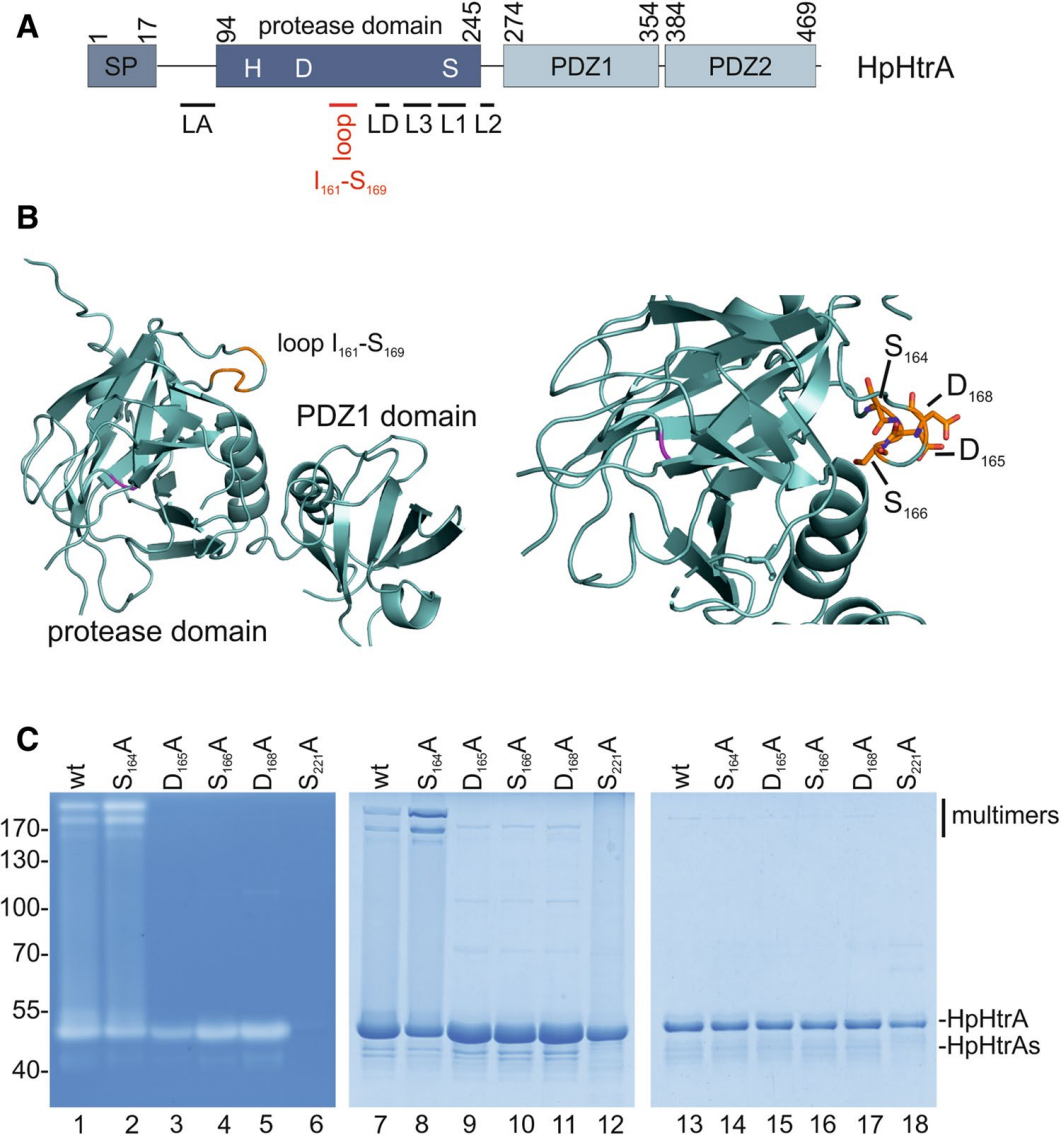
The allosteric ligand-binding loop is important for HtrA oligomer stabilization. (**A**) Model of HtrA showing the domain structure showing the signal peptide (SP), the extended linker region containing the LA loop, the protease domain with the catalytic triad histidine (H), aspartic acid (D), and serine (S), and the PDZ1 and PDZ2 domains. The protease domain also contains the regulatory LD, L1, L2, and L3 loops. The position of the ligand-binding loop is highlighted in red. (**B**) X-ray structure of the protease domain of HpHtrA^46^ with S_164_, D_165_, S_166_, and D_168_ indicated in the ligand-binding loop. S_221_ in the active center is highlighted in magenta. (**C**) Oligomerization and activity of HpHtrA wt, HpHtrA S_164_A, HpHtrA D_165_A, HpHtrA S_166_A, HpHtrA D_168_A, and HpHtrA S_221_A analyzed by casein zymography (lanes 1–6), non-reducing SDS-PAGE (lanes 7–12), and reducing SDS-PAGE (lanes 13–18).

**Figure 5:**
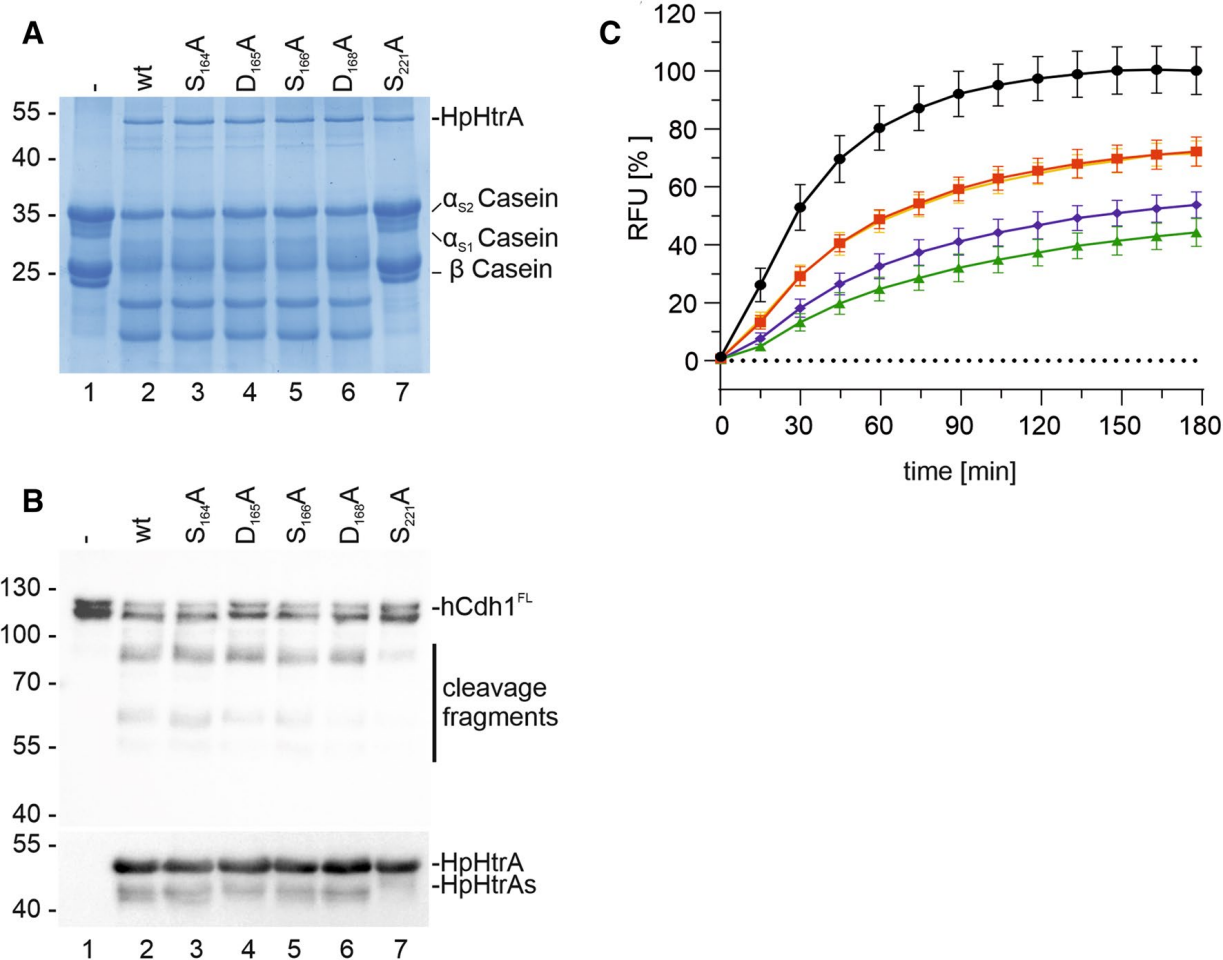
Proteolytic activity of HpHtrA loop mutants. To analyze the proteolytic activity of HtrA wt and its isogenic mutants HpHtrA S_164_A, HpHtrA D_165_A, HpHtrA S_166_A, HpHtrA D_168_A, and the inactive HpHtrA S_221_A, 10 µg casein (**A**) or 50 ng E-cadherin (hCdh1) (**B**) were incubated with 250 ng HtrA variants as indicated for 16 h at 37 °C in 50 mM HEPES buffer (pH 7.4). Cleavage of casein was analyzed by Coomassie-stained SDS-PAGE and hCdh1 cleavage was determined by Western blot analysis using an antibody recognizing the EC5 domain of hCdh1. HpHtrA and the auto-processed HpHtrAs were detected as indicated. (**C**) The activity of the ligand-binding loop mutants was analyzed using the FRET peptide as a substrate. 5 µM FRET peptide were incubated with 250 nM HpHtrA wt (black circle), HpHtrA S_164_A (red square), HpHtrA D_165_A (green triangle), HpHtrA S_166_A (yellow inverted triangles), and HpHtrA D_168_A (blue rhombus) for 180 min at 37 °C in 50 mM HEPES buffer (pH 7.4). The data represent the relative fluorescent units (RFU) ± S.D. with fluorescent signals obtained from FRET peptide treated with HpHtrA wt for 180 min set as 100%.

Oligomerization is required for the activation of the *E. coli* HtrA homologue DegP, which is converted into proteolytically active oligomers upon substrate binding^44^. HtrA proteases harbor several domains that are crucially important for assembly and activity. DegP/HtrA proteases contain an N-terminally located signal peptide (SP), followed by the protease domain, which is characterized by the catalytic triad histidine (H), aspartic acid (D), and serine (S). The C-terminally located domains PDZ1 and PDZ2 are important for substrate recognition and oligomerization^44^. Several regulatory loops (e.g. LA, LD, and L1–L3 loops) have been investigated for *E. coli* DegP, which share sequence similarities with HpHtrA (Fig. 4A, Fig. S6A, and Fig. S6B). An ‘orphan’ allosteric pocket (I_161_–S_169_) placed between the PDZ1 domain and the serine protease domain has been identified in the 2.6 Å X-ray structure of HpHtrA that functions as an important protein–protein interface (Fig. 4B and Fig. S6B)^45,46^. This allosteric pocket contains the amino acid motif S_164_, D_165_, S_166_, and D_168_, which is not only required for the stability and activity of HtrA oligomers^45^, but also for binding small molecule inhibitors that block HtrA activity^46^. Although typical Zn^++^- or Cu^++^-binding motifs characterized by histidine-rich regions are not present in this region, HpHtrA and, in particular, the allosteric loop may exhibit additional Zn^++^- and Cu^++^-binding sites composed of aspartic acid (D), glutamic acid (E), or cysteine (C), which can bind Zn^++^ or Cu^++^ equally well^47^. Therefore, we wanted to investigate whether Zn^++^ and Cu^++^ could target the aspartic acids exposed in this loop, which have previously been suggested as ligand-binding motifs^47,48^. The individual amino acids S_164_, D_165_, S_166_, and D_168_ were mutated to alanine and analyzed for oligomer stability (Fig. 4C). While HpHtrA S_164_A showed an increase in oligomer stability in casein zymography and SDS-PAGE under non-reducing conditions, oligomeric HpHtrA D_165_A, HpHtrA S_166_A, and HpHtrA D_168_A were completely disintegrated (Fig. 4C, left and middle panel), confirming that this loop has an important role in the allosteric regulation of HpHtrA. Proteolytically inactive HpHtrA S_221_A was included as a negative control and purity of recombinant HpHtrA proteins was demonstrated in an SDS-PAGE under denaturing conditions (Fig. 4C, right panel). Based on these data, we propose that D_165_, S_166_, and D_168_ in HpHtrA are not required for proteolytic activity, but rather for stabilization of the active conformation. The proteolytic activity of HpHtrA mutants was then analyzed in in vitro cleavage experiments. Even though the stability of all three HpHtrA oligomers was clearly diminished, casein (Fig. 5A) and E-cadherin hydrolysis (Fig. 5B) were only slightly changed compared to HpHtrA wt. We assume that the limited HtrA activity is sufficient to cleave the substrates within 16 h of incubation. In addition, the kinetics of substrate hydrolysis by HtrA mutants was investigated using the FRET peptide assay. The quantification revealed that mutation of S_164_ or S_166_ led to a decrease in proteolytic activity of approximately 30%. Mutation of D_165_ or D_168_ induced an even larger loss of HpHtrA activity (Fig. 5C), suggesting that mutations in the loop severely interfere with stability, oligomer formation, and/or activity. This observation is in agreement with our previous data, emphasizing the importance of this allosteric region. A bioactive ligand that was designed to bind to the allosteric pocket efficiently blocked the HtrA activity of *H. pylori* and *Campylobacter jejuni* and, subsequently, bacterial transmigration across the epithelial barrier^46^.

**Figure 6:**
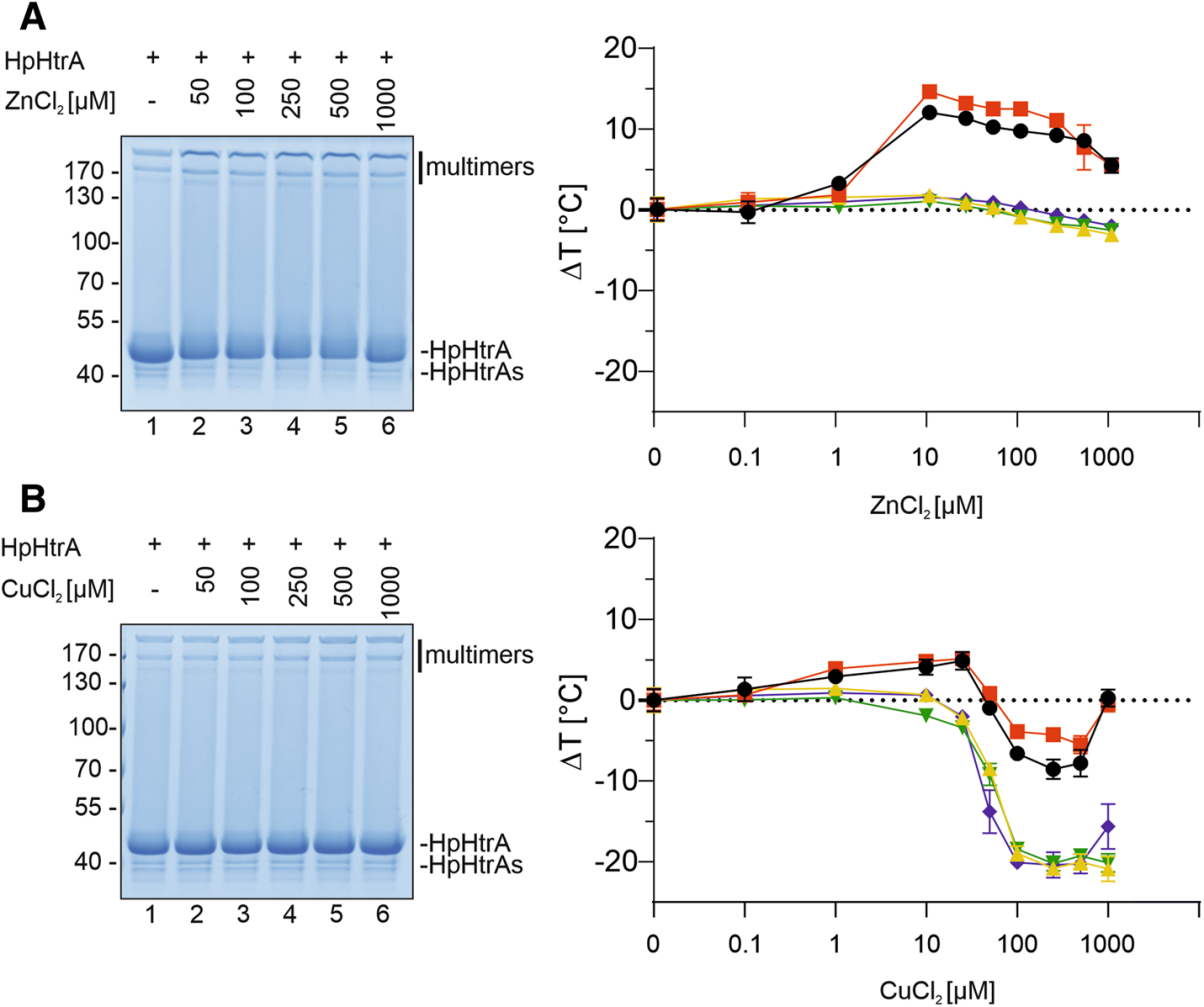
Zn^++^ and Cu^++^ ions interfere with the stability of HpHtrA via an allosteric loop. To investigate the effect of ZnCl_2_ and CuCl_2_ on the stability and activity of HpHtrA oligomers, HpHtrA wt was incubated with increasing concentrations of ZnCl_2_ (**A**) and CuCl_2_ (**B**) and then analyzed by a Coomassie-stained SDS-PAGE under non-reducing conditions (left panels). 4 µM recombinant HpHtrA wt (black circles), HpHtrA S_164_A (red squares), HpHtrA D_165_A (green inverted triangles), HpHtrA S_166_A (yellow triangles), and HpHtrA D_168_A (blue rhombus) were incubated with SYPRO Orange and increasing concentrations of ZnCl_2_ and CuCl_2_ at a temperature ramp from 25–95 °C (increase of 0.5 °C per minute). Changes in melting temperature ΔT_m_ are presented normalized to the intrinsic T_m_ of the individual HtrA mutant (right panels).

Since the allosteric loop is important for HpHtrA regulation and ligand binding, we further investigated whether Zn^++^ and Cu^++^ influence oligomerization of HpHtrA via this loop. HpHtrA proteins were treated with Zn^++^ and Cu^++^ and oligomerization was analyzed in Coomassie-stained protein gels under non-reducing conditions. In comparison to untreated HpHtrA wt, we observed a concentration-dependent effect of Zn^++^ addition on HpHtrA oligomerization. A Zn^++^ concentration of 50 µM was sufficient to enhance oligomerization or the stability of HpHtrA (Fig. 6A, left panel, lane 2), suggesting direct binding of Zn^++^ to HpHtrA. Using a thermal shift assay, we first determined the melting temperature of about 75 °C for HpHtrA wt, which is quite high and indicates an enhanced stability that enables survival under stress conditions^30,49^. Mutation of D_165_ and S_166_ resulted in an increase in the thermal denaturation of HpHtrA, while S_164_ and D_168_ mutations did not alter stability (Fig. S7). HpHtrA mutants were then incubated with Zn^++^. HpHtrA wt was stabilized at a concentration of approximately 10 µM Zn^++^. A similar effect was shown for HpHtrA S_164_A, indicating that Zn^++^ does not interfere with S_164_. However, Zn^++^ did not stabilize HpHtrA carrying mutations of D_165_, S_166_, or D_168_ (Fig. 6A, right panel). This could be explained either by the observation that HpHtrA D_165_A, HpHtrA S_166_A, and HpHtrA D_168_A mutants cannot form stable oligomers in general or by the assumption that Zn^++^ does not directly bind to the allosteric loop, but rather into the active pocket of HpHtrA, as it has been suggested for other serine proteases^33,36,37^. To answer this question, the binding of Zn^++^ was further analyzed by microscale thermophoresis (MST) (Table 1 and Fig. S8). Binding of Zn^++^ could be detected for HpHtrA wt (*K*_*d*_ = 23 µM) and HpHtrA S_164_A (*K*_*d*_ = 55 µM) with signal-to-noise ratios of 6.3 and 6.0, respectively. Although binding curves were also fitted for HpHtrA D_165_A, S_166_A, and D_168_A (Fig. S8), the signal-to-noise ratios were too low to prove binding of Zn^++^ to the proteins (Table 1). Cu^++^ was also investigated, but it strongly quenched the fluorescent signal at high concentrations and data analysis was therefore not possible (data not shown). Our results thus provide support for the hypothesis that HpHtrA D_165_ and D_168_ are involved in the interaction with Zn^++^ as a putative binding site. Since the mechanism of HpHtrA activity has not yet been intensively investigated, it is still unknown whether oligomerization is required for proteolytic activity or whether HpHtrA forms active oligomers in the zymogram after renaturation. But based on our data in this report, we conclude that the allosteric loop is important for the stability of oligomeric HpHtrA. Interaction with Zn^++^ might drastically change the conformation of HpHtrA, leading to an increase in oligomer stability, but also to an efficient inhibition of the proteolytic activity or substrate recognition. The detailed mechanism of HpHtrA oligomerization and activity will be further investigated in the future.

**Table 1:** HpHtrA interaction with Zn^++^.

Protein	Response amplitude	S/N^a^	*K*_*d*_^b^ (µM ± SD)
HpHtrA wt	4.3	6.3	23 ± 12
HpHtrA S_164_A	3.6	6.0	55 ± 34
HpHtrA D_165_A	3.6	3.9	n.d.^c^
HpHtrA S_166_A	3.9	3.8	n.d
HpHtrA D_168_A	2.7	3.5	n.d

^a^S/N, signal to noise ratio; ^b^*K*_*d*_, dissociation constant; ^c^n.d., not determined.

The data obtained from experiments with Cu^++^ differ considerably from those obtained with Zn^++^. HpHtrA wt was only slightly stabilized at low Cu^++^ concentrations, as analyzed by Coomassie-stained SDS-PAGE under non-reducing conditions (Fig. 6B, left panel). Comparing the HpHtrA mutants in the sensitive thermal shift assay, two modes of regulation were observed. At low concentrations, Cu^++^ stabilized HpHtrA wt and HpHtrA S_164_A, but not the HpHtrA D_165_A, HpHtrA S_166_A, and HpHtrA D_168_A mutants (Fig. 6B, right panel), resembling the effects detected in response to Zn^++^ (Fig. 6A, right panel). Higher concentrations of Cu^++^ finally destabilized HpHtrA wt and HpHtrA S_164_A. Interestingly, the HpHtrA D_165_A, HpHtrA S_166_A, and HpHtrA D_168_A mutants were more strongly destabilized by Cu^++^ (Fig. 6B, right panel), indicating that these sites in HpHtrA are not only involved in oligomer stability, but also directly targeted by Cu^++^. In conclusion, we assume that Cu^++^ has two binding motifs in the HpHtrA molecule. The first is the active site, which is a high affinity binding site for Zn^++^ and Cu^++^ and can bind the metals already at lower concentrations. This has been suggested for a number of serine proteases that bind Zn^++^ and Cu^++^ via the histidine in their pockets^33,36,37^. The second motif in the HpHtrA molecule is a low affinity binding site formed by the allosteric loop, the binding of which leads to a destabilization of HpHtrA. However, the detailed mechanism of Zn^++^ and Cu^++^ interference with HpHtrA needs further attention and will be investigated in future studies.

## Conclusion

The significance of HtrA proteins in bacterial pathogenesis is well established. Therefore, potent inhibitors are desired to block *H. pylori* pathogenesis. First lead structures have been developed and successfully tested in in vitro systems^28,29,46^. In this study, we developed a sensitive FRET peptide assay for measurement of HpHtrA activity that is suitable for high-throughput screenings. As a paradigm for serine protease inhibition, we tested divalent ions as putative HpHtrA protease inhibitors and found Zn^++^ and Cu^++^ as inhibitory ligands. Although HpHtrAs do not require binding of metal ions for their regulation, Zn^++^ ions have been described to block serine protease activity through binding to the active center^33^. Therefore, Zn^++^, and possibly Cu^++^, has the capacity to potentiate the inhibitory effects of moderate HtrA inhibitors, as it has previously been demonstrated for serine proteases. The development of a Zn^++^- and/or Cu^++^-mediated serine protease inhibitor functioning in a low nanomolar range is a stated objective in *H. pylori* research, since efficient inhibition of HpHtrA prevents bacterial pathogenesis and significantly attenuates *H. pylori* growth and survival.

## Methods

### Recombinant proteins

Recombinant human E-cadherin (hCdh1, D155-I707, accession no. NP_004351) was obtained from Sino Biological (Vienna, Austria). According to the manufacturer’s protocol, the lyophilized hCdh1 was reconstituted in sterile water to give a concentration of 250 ng/ml. Casein was obtained from Carl Roth (Karlsruhe, Germany) and reconstituted in water. Purification of HtrA wild type (wt) from the *H. pylori* strain Hp26695 (HpHtrA, G18-K475, UniProt G2J5T2), its isogenic inactive mutant (HpHtrA S_221_A), as well as HpHtrA mutants S_164_A, D_165_A, S_166_A, and D_168_A was performed as previously described^40^. Briefly, transformed *E. coli* BL21 were grown in terrific broth (TB) medium to an OD_600_ of 0.7 and expression of glutathione S-transferase (GST)-HtrA was induced by addition of 100 µM isopropyl β-d-1-thiogalactopyranoside (IPTG) for 3 h at 30 °C. The bacteria were pelleted and lysed by sonication in phosphate-buffered saline (PBS). The lysate was cleared by centrifugation and recombinant GST-tagged HpHtrA proteins were bound to glutathione sepharose beads (GE Healthcare Life Sciences, Vienna, Austria). The GST tag was removed by addition of PreScisssion protease (GE Healthcare Life Sciences, Vienna, Austria) for 16 h at 4 °C. After purification, the proteins were dialyzed against 50 mM tris(hydroxymethyl)aminomethane (Tris)/HCl (pH 7.5) and 150 mM NaCl. Purity of proteins was routinely determined by sodium dodecyl sulfate polyacrylamide gel electrophoresis (SDS-PAGE) and staining with Coomassie Brilliant Blue G250 (Carl Roth, Karlsruhe, Germany).

### DIPPS

Direct in-gel profiling of protease specificity (DIPPS) has been described previously^35^. Briefly, 150 µg of the cellular proteome of MKN-28 cells was separated by SDS-PAGE and stained with Coomassie Brilliant Blue G250. Protein lanes were sliced, cut into pieces, and destained twice with 150 μl 50% acetonitrile in 25 mM ammonium bicarbonate for 30 min at 25 °C and 1,200 rpm. The gel pieces were dehydrated with 100% acetonitrile and vacuum‐dried. The dehydrated pieces were incubated with 1 µM HpHtrA in 100 mM Tris (pH 8.0) and 50 mM NaCl for 2 h at 37 °C. Following in‐gel proteolysis, the generated peptides were extracted from the gel and analyzed by LC‐MS/MS using an Orbitrap LTQ Velos mass spectrometer coupled to EASY-nanoLC II HPLC unit (Thermo Fischer Scientific). Database searches were performed using the MaxQuant software package version 1.6.3.4^50,51^ and UniProt derived human reference proteome (UniProtKB, *Homo sapiens*, canonical database containing 20,402 entries, released in February 2019), as described previously^35^. The cleaved peptides were identified with a length‐limited unspecific database search and aligned to generate a substrate specificity profile of the studied protease. Visualization of relative ratios as iceLogos^52^ provided unambiguous insight into the extended cleavage preferences of HpHtrA.

### FRET peptide assay

For the establishment of a FRET-based assay for the quantification of the HpHtrA activity, the 2-Abz/3-nitrotyrosine FRET pair^53–55^ was introduced into the sequence AQRVAF: the fluorophore (2-Abz, Ex/Em 320/435 nm) was coupled at the N-terminus, whereas the quencher (3-nitrotyrosine, Ex 425 nm^56^) was positioned at the C-end. The FRET activity of this peptide was first confirmed by measuring the fluorescence emission in the range 400–450 nm before and after the trypsin-mediated cleavage of the peptide bond R_V (Fig. S3). The measurements were performed in a black, flat-bottom 96-well plate (Nunc, Thermo Scientific, Schwerte, Germany) at 37 °C. 5 µM of the peptide were incubated with 250 nM HpHtrA and 125 nM trypsin in 50 mM HEPES buffer (pH 7.4). Where indicated, increasing concentrations of ZnCl_2_ or CuCl_2_ were added. Cleavage of the peptide leads to increasing fluorescence, which was measured in a plate reader (Infinite 200 PRO, TECAN, Groedig, Austria) with a filter setting of 320 nm/425 nm (Ex/Em). Statistical analysis was performed with GraphPad Prism software (Vers. 8.0.2). One-way ANOVA was used to statistically compare changes in fluorescence between samples treated with HpHtrA wt or HpHtrA S_221_A with those treated with trypsin or with increasing concentrations of ZnCl_2_ or CuCl_2_, or the untreated control. Three independent experiments containing three technical replicates were analyzed for every sample. Significance is indicated as non-significant (ns) for *p* > 0.05, * for *p* < 0.05, ** for *p* < 0.01, *** for *p* < 0.001, and **** for *p* < 0.0001.

### In vitro cleavage experiments, SDS-PAGE, and Western blot

For in vitro cleavage experiments, 10 µg casein were incubated with 250 ng HpHtrA wt (MW 50,000) or inactive mutant in 20 µl 50 mM HEPES buffer (pH 7.4) for 16 h at 37 °C. Where indicated, 1 mM of CaCl_2_, MgCl_2_, MnCl_2_, ZnCl_2_, BaCl_2_, CuCl_2_, NiCl_2_, CoCl_2_, ethylenediaminetetraacetic acid (EDTA), or ethylene glycol-bis(β-aminoethyl ether)-*N*,*N*,*N*′,*N*′-tetraacetic acid (EGTA) were added to the cleavage reactions. The proteins were separated by SDS-PAGE and visualized by staining with Coomassie Brilliant Blue G250 (Lactan, Vienna, Austria). Alternatively, 50 ng recombinant hCdh1 were incubated with 250 ng HpHtrA wt or inactive mutant for 16 h at 37 °C in combination with different chlorides as indicated. Proteins were separated using SDS-PAGE and blotted onto a nitrocellulose membrane. Human Cdh1 was detected by an antibody recognizing the EC5 domain of hCdh1 (Abcam, Cambridge, UK) and a polyclonal serum was used to detect HpHtrA^22^. Independent experiments were repeated at least three times.

### Casein zymography and non-reducing and reducing SDS-PAGE

HpHtrA wt was incubated with 1 mM of CaCl_2_, MgCl_2_, MnCl_2_, ZnCl_2_, BaCl_2_, CuCl_2_, NiCl_2_, CoCl_2_, EDTA, or EGTA in 50 mM HEPES buffer (pH 7.4) for 60 min on ice. Non-reducing sample buffer without β-mercaptoethanol was added and the samples were separated by SDS-PAGE containing 0.1% casein (Carl Roth, Karlsruhe, Germany) as a substrate. The gel was incubated for 60 min in renaturing buffer (2.5% Triton X-100) and equilibrated for 30 min in developing buffer (50 mM Tris–HCl, pH 7.4, 200 mM NaCl, 5 mM CaCl_2_, and 0.02% Brij-35) at room temperature under gentle agitation. Afterwards, the gel was incubated for 24 h at 37 °C in developing buffer under gentle agitation. To visualize caseinolytic activity, the gel was stained in 0.5% Coomassie Brilliant Blue R250 (Carl Roth, Karlsruhe, Germany). For detection of multimers by SDS-PAGE, HpHtrA was separated by SDS-PAGE under non-reducing conditions. Proteins were visualized by staining with Coomassie Brilliant Blue G250. Independent experiments were repeated at least four times.

### Thermal shift assay

Thermal shift assays were performed to determine the melting temperatures of recombinant HpHtrA proteins (wt, S_164_A, D_165_A, S_166_A, D_168_A) in the presence of increasing concentrations of ZnCl_2_ or CuCl_2_. A change in the melting temperature in the presence ZnCl_2_ or CuCl_2_ indicates stabilization or destabilization of the protein and is therefore evidence for binding of Zn^++^ or Cu^++^ to HpHtrA. Recombinant HpHtrA proteins (wt, S_164_A, D_165_A, S_166_A, D_168_A) in 50 mM HEPES buffer (pH 7.4) were mixed with SYPRO Orange (Sigma-Aldrich, Vienna, Austria) to final concentrations of 4 µM HpHtrA and 7 × SYPRO Orange in a 96-well PCR plate. Where indicated, increasing concentrations of ZnCl_2_ and CuCl_2_ were added. Fluorescence data was collected using an Applied Bioscience StepOne Plus PCR cycler (Thermo Fisher Scientific, Schwerte, Germany) during a temperature ramp from 25–95 °C (0.5 °C per min) with a 4 min hold at 25 °C and 95 °C. SYPRO Orange binds to exposed hydrophobic regions within the denatured HpHtrA proteins, which increases the fluorescence of the dye^57,58^. The fluorescence emission signal can then be used to determine the melting temperature of the protein, which is located at the infliction point of the fluorescence signal curve. The experiment was performed in triplicates.

## Supplementary information

Supplementary file1 (PDF 2020 kb)

## Data Availability

The datasets generated and analyzed during the current study are available from the authors on request.
