# Supplementary material for: A novel FRET peptide assay reveals efficient Helicobacter pylori HtrA inhibition through zinc and copper binding
Source: Sci Rep. 2020 Jun 29;10:10563. doi: 10.1038/s41598-020-67578-2 (PMC7324608; doi:10.1038/s41598-020-67578-2)
Supplement: Supplementary file 1 — Supplementary file1 (PDF 2020 kb) [file 41598_2020_67578_MOESM1_ESM.pdf]

A novel FRET peptide assay reveals efficient *Helicobacter pylori* HtrA inhibition through zinc and copper binding

Sabine Bernegger[1], Cyrill Brunner[2], Matej Vizovišek[3], Marko Fonovic[3], Gaetano Cuciniello[1,#], Flavia Giordano[4,§], Vesna Stanojlovic[4], Miroslaw Jarzab[1], Philip Simister[5], Stephan M. Feller[6], Gerhard Obermeyer[7], Gernot Posselt[1], Boris Turk[3], Chiara Cabrele[4], Gisbert Schneider[2], Silja Wessler[1*]

Supplementary information:

Methods . . . . Page S2

Figure S1 . . . . Page S4

Figure S2 . . . . Page S8

Figure S3 . . . . Page S9

Figure S4 . . . . Page S10

Figure S5 . . . . Page S11

Figure S6 . . . . Page S12

Figure S7 . . . . Page S13

Figure S8 . . . . Page S14

Original blots for Fig. 2 . . . . Page S15

Original blots for Fig. 3 . . . . Page S16

Original gels and blots for Fig. 4 . . . . Page S17

Original blots for Fig. 5 . . . . Page S18

Original gels and blots for Fig. 6 . . . . Page S19

References . . . . Page S20

Methods

Chemicals for peptide synthesis and analytics. Chemical reagents and solvents for the peptide synthesis were of peptide-synthesis grade. The natural Fmoc-protected amino acids, Fmoc-3-nitro-tyrosine, Rink-amide MBHA resin (loading 0.57 mmol/g), N,N-dimethylformamide (DMF), N-methyl-2-pyrrolidone (NMP), dichloromethane (DCM), diethylether, and N,N-diisopropylethylamine (DIPEA) were purchased from Iris Biotech (Marktredwitz, Germany). 2-(1H-benzotriazole-1-yl)-1,1,3,3-tetramethyluronium hexafluorophosphate (HBTU), N-hydroxybenzotriazole (HOBt), piperidine, and trifluoroacetic acid (TFA) were purchased from Biosolve (Valkenswaard, The Netherland). Acetonitrile (ACN), triisopropylsilane (TIS), 1,2-ethanedithiol (EDT), thioanisole (TIA), and 2-(Boc-amino)-benzoic acid (2-Boc-Abz-OH) were purchased from Sigma-Aldrich (Vienna, Austria). HPLC-grade TFA was from Alfa-Aesar (Karlsruhe, Germany). α-Cyano-4-hydroxycinnamic acid (CHCA) was purchased from Acros Organics (Vienna, Austria). Trypsin was purchased from AppliChem (Darmstadt, Germany).

Peptide synthesis and analytics. The solid-phase peptide synthesis was performed manually by using Fmoc-chemistry. HPLC analysis was carried out on a Dionex UltiMate 3000 system from Thermo Fisher Scientific (Germering, Germany), equipped with a Syncronis C-18 column (100 Å, 5 µm, 250x4.6 mm, Thermo Fisher Scientific), and a diode-array detector set at 220 nm. The binary elution system consisted of (A) 0.06% (v/v) TFA in water, and (B) 0.05% (v/v) TFA in ACN (flow rate: 1.5 ml/min). The following gradient was used: 3% B for 8 min, 3-60% B over 35 min. MALDI-TOF mass spectra were recorded on an Autoflex mass spectrometer (Bruker Daltonics, Bremen, Germany) by using CHCA as matrix. For the determination of the peptide concentration in 50 mM sodium phosphate buffer (pH 7.3), the UV spectrum was recorded on an Agilent Cary 60 UV-Vis spectrophotometer (the buffer was used as blank). The absorbance of the 3-nitro-tyrosine residue at 381 nm and a molar extinction coefficient of 2200 M^{-1} cm^{-1} were used to calculate the peptide concentration[1]. To prove the intramolecular fluorescence quenching, fluorescence measurements of the peptide in absence and presence of trypsin or HpHtrA were performed on an Agilent Cary Eclipse fluorescence spectrophotometer.

Synthesis of 2-Abz-AQRVAFY(3-NO_2)-NH_2. The peptide was assembled manually on a 20 µmol scale. The side-chain protecting groups were Pbf for Arg, and Trt for Gln. Fmoc deprotection was carried out with 25% piperidine in DMF/NMP (80:20, v/v) for 3 min, and 12.5% piperidine in DMF/NMP (80:20, v/v) for 12 min. The double couplings (2x45 min) were accomplished with the mixture Fmoc-AA-OH/HOBt/HBTU/DIPEA (5:5:4.8:10 equiv.). 2-Boc-Abz-OH was coupled with the same protocol. Afterwards, the peptidyl-resin was treated with 25% piperidine in DMF/NMP (80:20, v/v) (2x12 min) to deacylate the 3-nitro-tyrosine side chain. The peptide was simultaneously cleaved from the resin and deprotected by using the mixture TFA/H_2O/TIA/EDT/TIS (90:3:1:3:3; V_{tot} = 1 ml) for about 3 h, precipitated from ice-cold diethyl ether, recovered by centrifugation at 4 °C for 6 min, washed four times with ice-cold diethyl ether, and dried in vacuo. The solid was dissolved in water and lyophilized. HPLC: 28.4 min (90%). MALDI-TOF-MS (negative mode): 1016.03 Da, found for $(M-H)^-$ (theoretical mass for $C_{47}H_{64}N_{14}O_{12}$: 1017.12 Da).

Microscale thermophoresis. In order to determine binding of Zn^{++} to HpHtrA wt, S_{164}A, D_{165}A, S_{166}A and D_{168}A, MicroScale Thermophoresis (MST) analysis was performed using the Monolith NT.115 (NanoTemper Technologies). HpHtrA proteases were labeled with red fluorescent dye NT-650-NHS (amine reactive) according to the manufacturer's protocol. Remaining free dye was removed by buffer exchange column chromatography and proteins were eluted in 50 mM Tris pH 7.4, 150 mM NaCl, 0.04% Tween. 5 nM of labelled HpHtrA was incubated for 15 minutes at RT with decreasing concentrations of $ZnCl_2$ (1 mM - 30.5 nM in a 1:1 dilution series). Measurement was performed with premium treated capillaries (NanoTemper Technologies) and the Monolith NT.115 Microscale Thermophoresis device. Data analysis was performed with MO.Affinity Analysis v2.3 (NanoTemper Technologies) and GraphPad Prism Software (Version 8.0.2). Three independent experiments were performed.

Figure S1

A

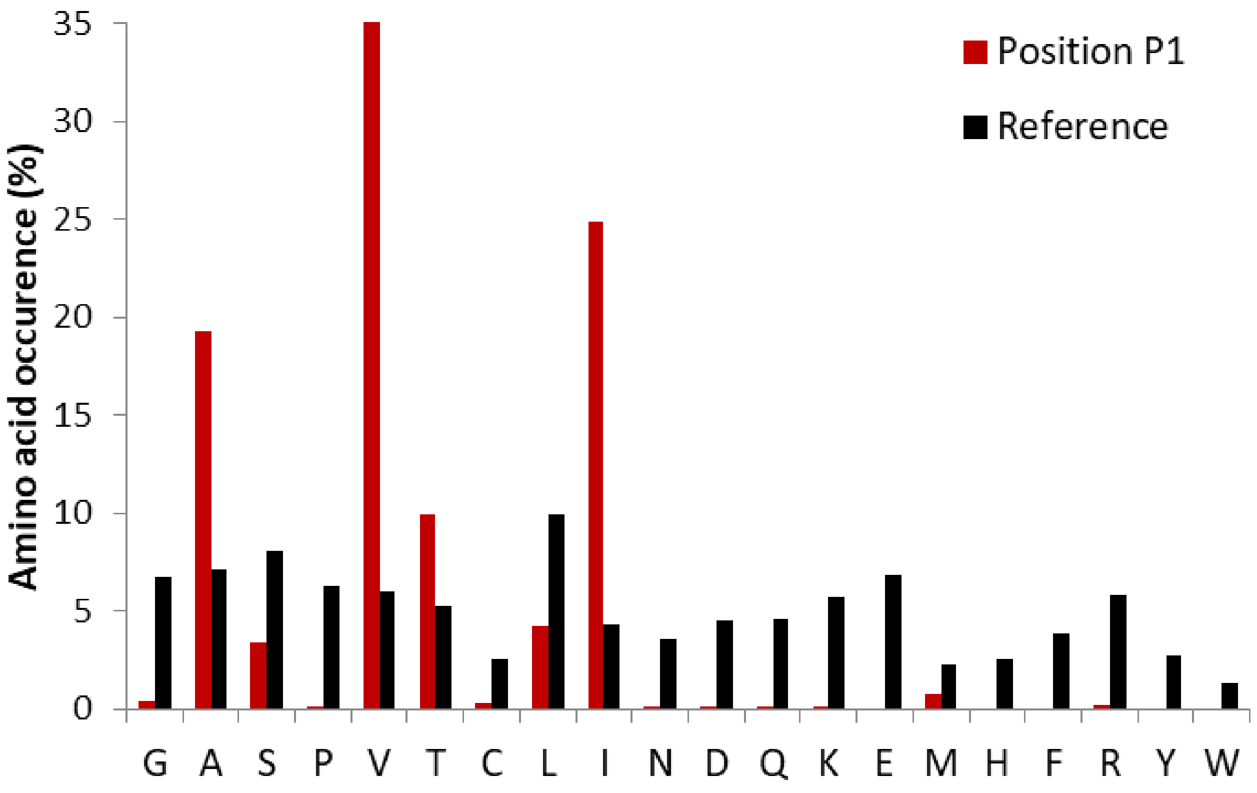

Position P1
Reference
Amino acid occurence (%)
35
30
25
20
15
10
5
0
G A S P V T C L I N D Q K E M H F R Y W

B

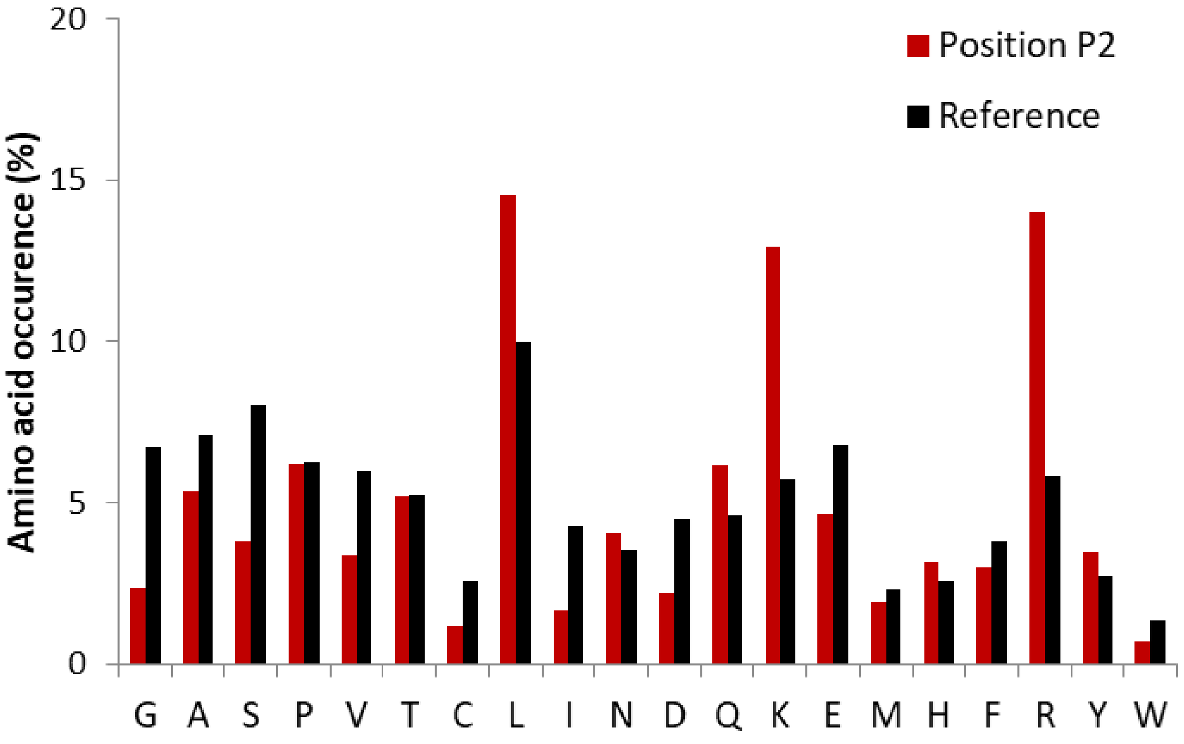

Position P2
Reference
Amino acid occurence (%)
20
15
10
5
0
G A S P V T C L I N D Q K E M H F R Y W

C

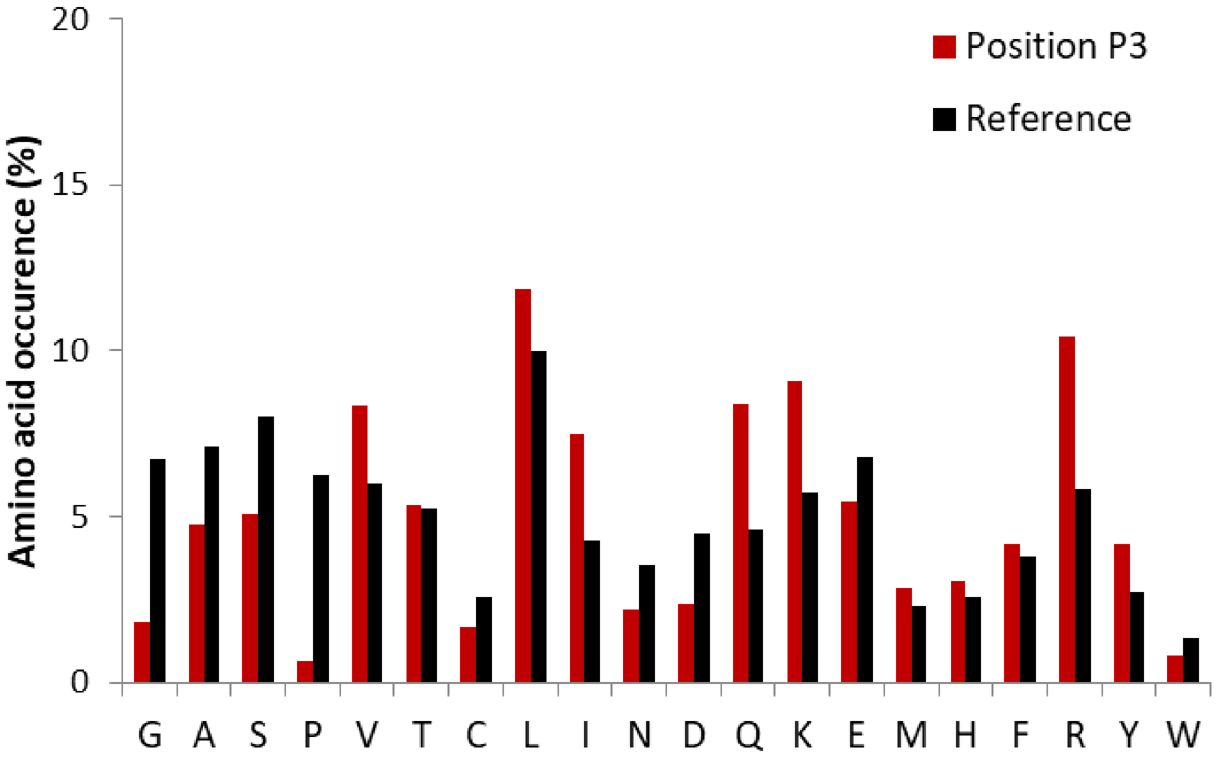

20
15
10
5
0
Amino acid occurence (%)
Position P3
Reference
G A S P V T C L I N D Q K E M H F R Y W

D

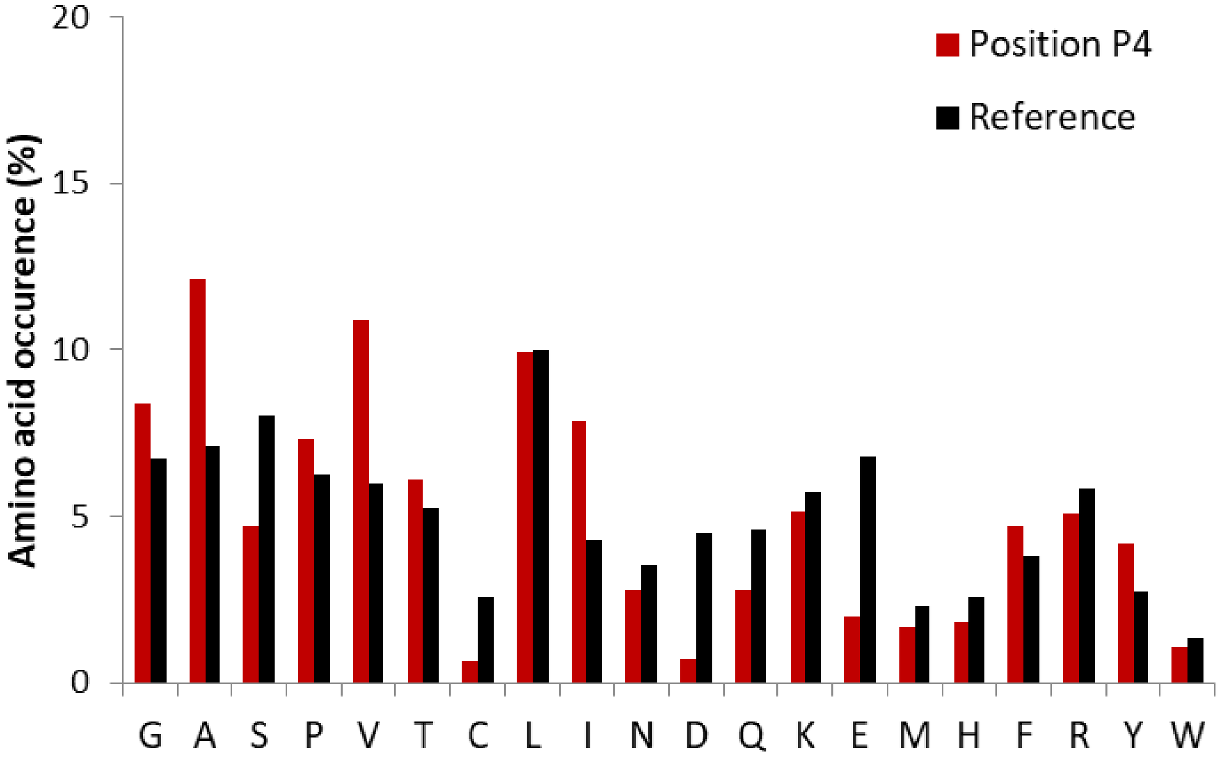

20
15
10
5
0
Amino acid occurence (%)
Position P4
Reference
G A S P V T C L I N D Q K E M H F R Y W

E

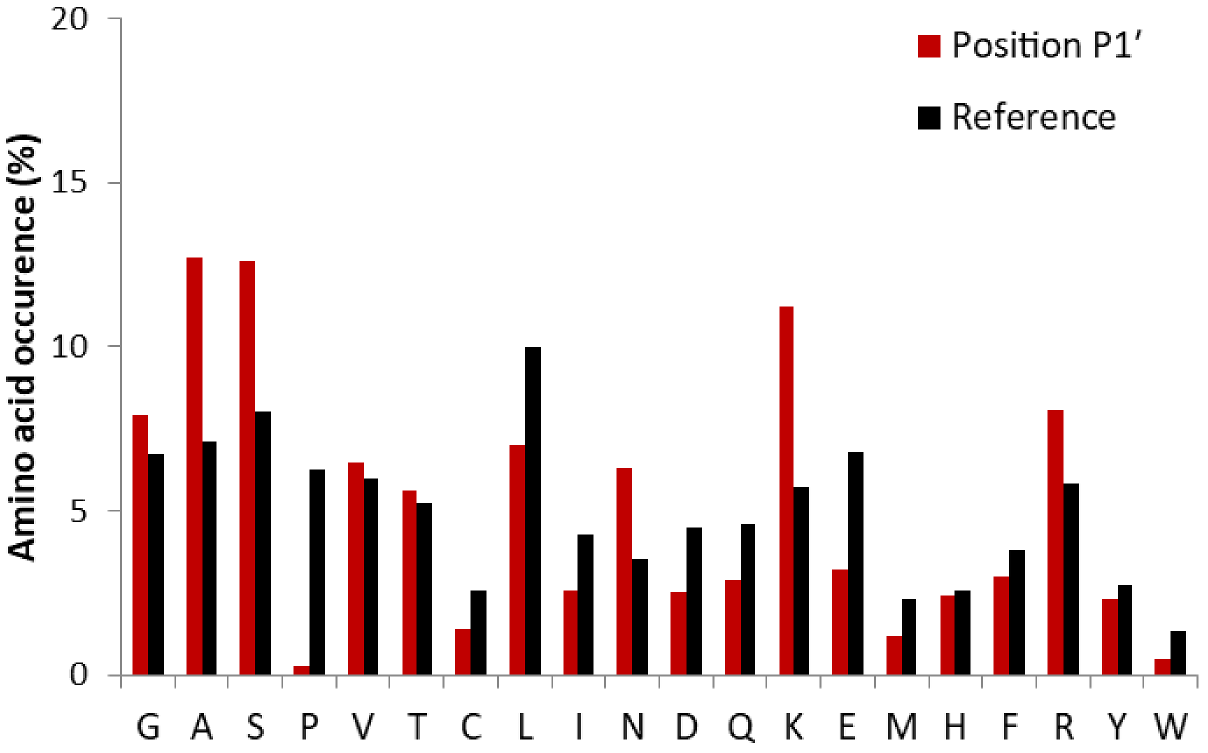
Position P1′
Reference
Amino acid occurence (%)
20
15
10
5
0
G A S P V T C L I N D Q K E M H F R Y W

F

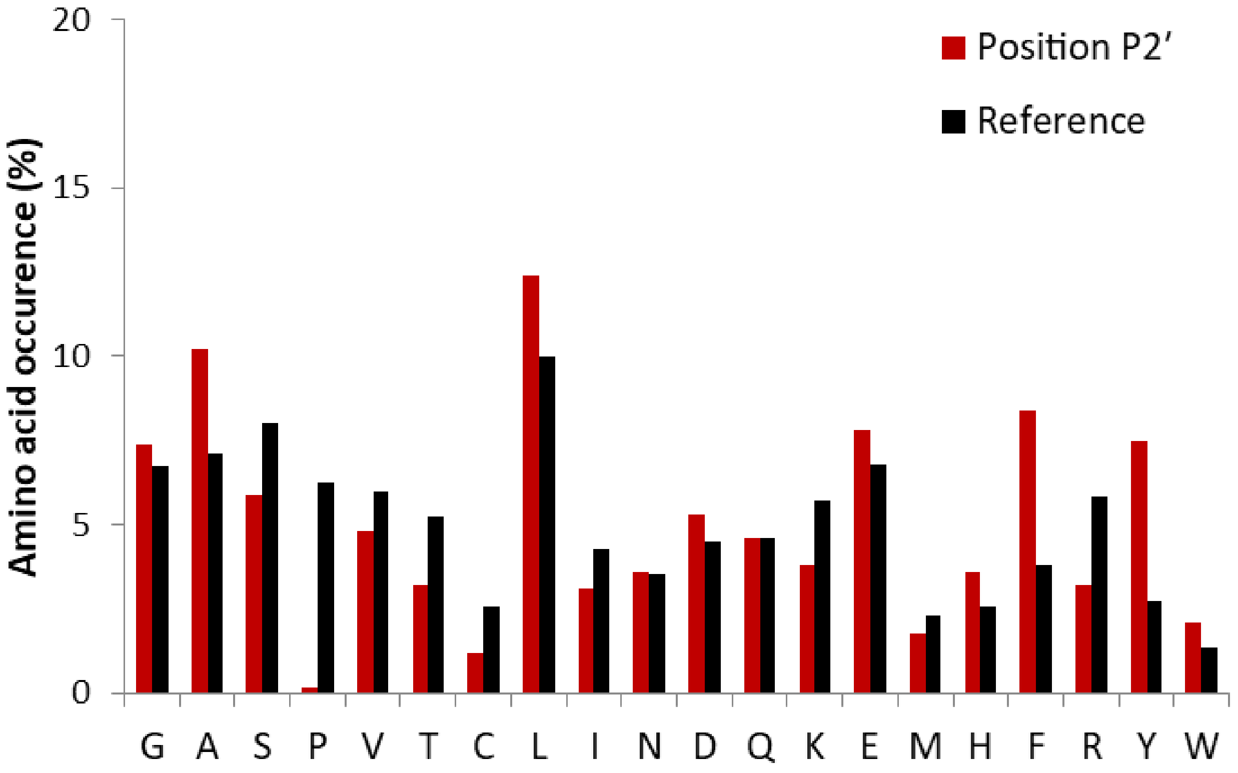
Position P2′
Reference
Amino acid occurence (%)
20
15
10
5
0
G A S P V T C L I N D Q K E M H F R Y W

Fig. S1 HpHtrA cleavage specificity profiling. The amino acid frequency distribution of P4 to P2’ sites of cleaved peptides is shown in comparison with the human proteome amino acid frequency distribution. HpHtrA cleavage preference are shown for the site P1 (A), P2 (B), P3 (C), P4 (D), P1´ (E), and P2´ (F).

Figure S2

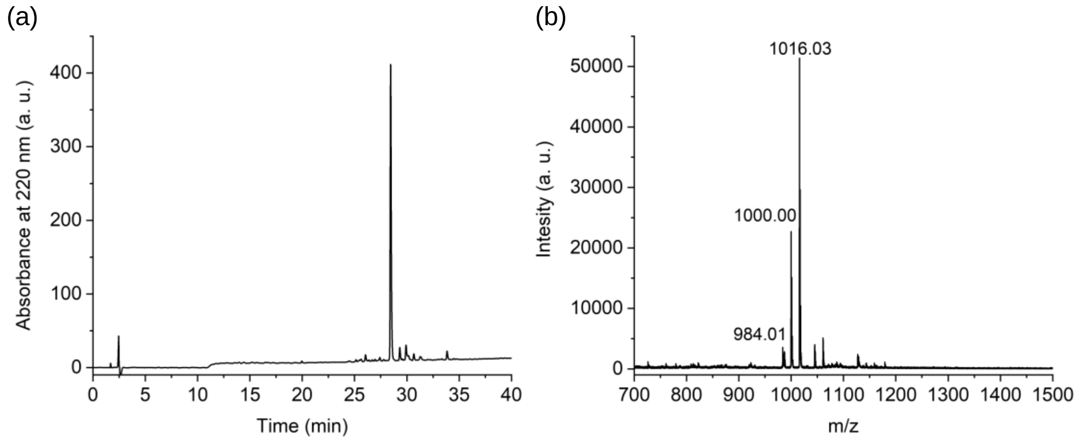

Fig. S2. Analytical HPLC (a) and MALDI-TOF-MS (negative mode) (b) of 2-Abz-AQRVAF-Y(3-NO_2)-NH_2.

The MS spectrum shows the characteristic MS fragmentation of the nitro group (-16 Da, -32 Da).

Figure S3

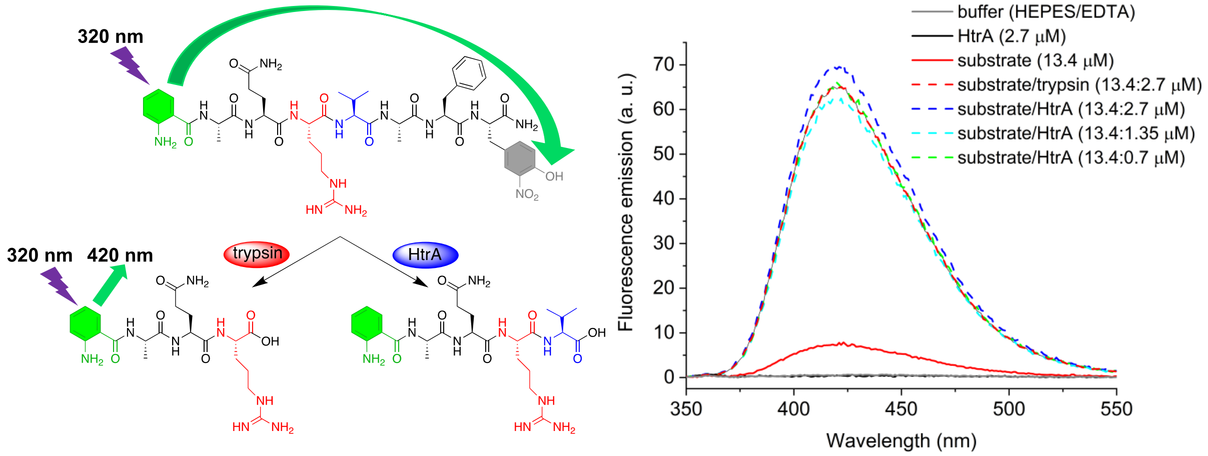

Fig. S3. Intramolecular fluorescence quenching of the fluorogenic peptide 2-Abz-AQRVAF-Y(3-NO_2)-NH_2. To prove the intramolecular quenching of the 2-Abz fluorescence by 3-nitro-tyrosine, 120 µl of a peptide solution (56 µM in 50 mM phosphate buffer, pH 7.3) were diluted with 380 µl of 50 mM HEPES/EDTA buffer (pH 7.4) to a final peptide concentration of 13.4 µM. The peptide/protease mixtures were prepared by mixing 120 µl of the peptide solution with trypsin or HpHtrA solutions: the final peptide concentration was 13.4 µM, whereas the final protease concentrations were 2.7 µM for trypsin, and 0.7 µM, 1.35 µM, and 2.7 µM for HpHtrA. All samples were incubated overnight at 37°C. Then, the fluorescence emission spectra were recorded upon excitation at 320 nm at 24°C. A fluorescence emission maximum for the 2-Abz moiety was observed at 420 nm, which showed ten times higher intensity in the presence of the protease, thus confirming the intramolecular quenching activity of 3-nitro-tyrosine on the 2-Abz group. No significant changes were observed between trypsin and HpHtrA or between the different HpHtrA concentrations.

Figure S4

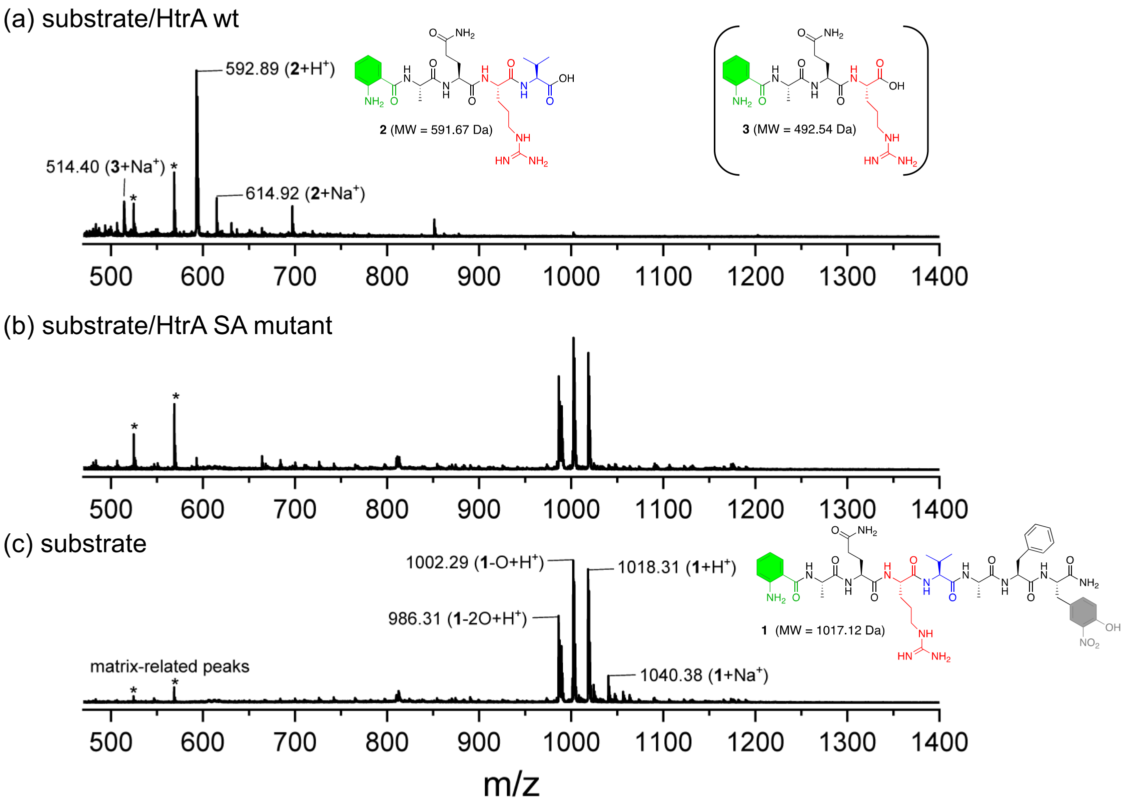

Fig. S4. MS analysis of the HpHtrA-mediated cleavage products of 2-Abz-AQRVAF-Y(3-NO_2)-NH_2. The peptide was incubated alone or in the presence of HpHtrA wt or the inactive SA mutant at the 20:1 ratio in 50 mM HEPES buffer (pH 7.4) for 3 h at 37°C. The samples were desalted by using ZipTip® pipette tips (Merck Millipore) and measured by MALDI-TOF-MS (positive mode). The peptide was found to be stable in the buffer in the absence of the protease as well as in the presence of the HpHtrA SA mutant. Instead, it was fully cleaved, preferentially after Val, by HpHtrA.

Figure S5

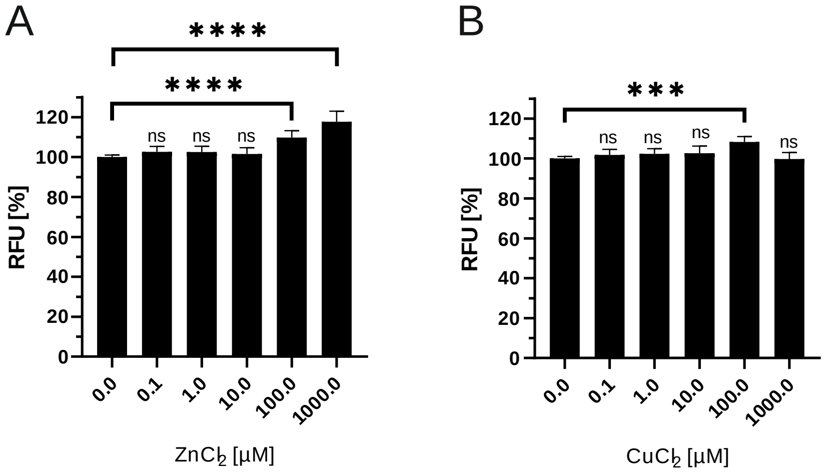

Fig. S5. Divalent ions do not quench fluorophores. The peptide was incubated with HpHtrA for 180 min at 37°C at a ratio of 20:1 in 50 mM HEPES buffer (pH7.4). The cleaved peptide was aliquoted and incubated with increasing concentrations of $ZnCl_2$ or $CuCl_2$ ranging from 0.1-1000 µM. After incubation for 30 minutes at RT in the dark, fluorescence of the cleaved peptides was measured. The data represent the relative fluorescent units (RFU) ± S.D. with fluorescent signals obtained from HpHtrA wt treated FRET peptide set as 100%. Asterisks indicate statistically significant differences (***$p=0.001$; ****$p < 0.0001$; ns, non-significant).

Figure S6

A

```
CLUSTAL O(1.2.4) multiple sequence alignment

TR|G2J5T2|G2J5T2_HELPY MKKTLFISLALALSLNAGNIQIQSMPKVKERVSVPSKDDTIYSYHDSIKDSIKAVVNIST 60
SP|P0C0V0|DEGP_ECOLI   MKKTTLALSALALSLGLALSPLSA---TAAETSSATTAQQMPSLAPMLEKVMPSVVSINV 57
                       **** :   ******. .  :.:    .   ..*   :. : : *     ::. : :**.*..
                                         LA
TR|G2J5T2|G2J5T2_HELPY EK-------KIKN---NFIG---------GGVFNDPFFQQFFGDLGGMIPKERMERALGS 101
SP|P0C0V0|DEGP_ECOLI   EGSTTVNTPRMPRNFQQFFGDDSPFCQEGSPFQSSPFCQGGQGGNGGGQ--QQKFMALGS 115
                       *        :: .   :*:*          . . ..** *    *. **     ::   ****

TR|G2J5T2|G2J5T2_HELPY GVIIS-KDGYIVTNNHVIDGADKIKVTIPGSNKEYSATLVGTDSESDLAVIRITK-DNLP 159
SP|P0C0V0|DEGP_ECOLI   GVIIDADKGYVVTNNHVVDNATVIKVQLSD-GRKFDAKMVGKDPRSDIALIQIQNPKNLT 174
                       ****. ..**:******:*.*   *** : . .:::.*.:**.* .**:*:*:* :  .**
                                                 LD               L3              L1
TR|G2J5T2|G2J5T2_HELPY TIKFSDSNDISVGDLVFAIGNPFGVGESVTQGIVSALNKSGIGINSYENFIQTDASINPG 219
SP|P0C0V0|DEGP_ECOLI   AIKMADSDALRVGDYTVAIGNPFGLGETVTSGIVSALGRSGLNAENYENFIQTDAAINRG 234
                       :**::**: : *** ..*******:**:**.******.:**:. :.***********:** *

TR|G2J5T2|G2J5T2_HELPY NSGGALIDSRGGLVGINTAIISKTGGNHGIGFAIPSNMVKDTVTQLIKTGKIERGYLGVG 279
SP|P0C0V0|DEGP_ECOLI   NSGGALVNLNGELIGINTAILAPDGGNIGIGFAIPSNMVKNLTSQMVEYGQVKRGELGIM 294
                       *******:: .* *:*******::   *** ************: .:*::: *:::** **:
                                                L2
TR|G2J5T2|G2J5T2_HELPY LQDLSGDLQNS--YDNKEGAVVISVEKDSPAKKAGILVWDLITEVNGKKVKNTNELRNLI 337
SP|P0C0V0|DEGP_ECOLI   GTELNSELAKAMKVDAQRGAFVSQVLPNSSAAKAGIKAGDVITSLNGKPISSFAALRAQV 354
                         :*..:* ::    * :.**.* .*   :* * **** . *:**.:*** :..    **  :

TR|G2J5T2|G2J5T2_HELPY GSMLPNQRVTLKVIRDKKERAFTLTLAERKNPNKKETISAQNGAQGQLNGLQVEDLTQET 397
SP|P0C0V0|DEGP_ECOLI   GTMPVGSKLTLGLLRDGKQVNVNLELQQSSQNQ-VDS-------SSIFNGIEGA------ 400
                       *:*   ..::** ::** *:   ..* * : .: :  ::        .. :**::

TR|G2J5T2|G2J5T2_HELPY KRSMRLSDDVQGVLVSQVNENSPAEQAGFRQGNIITKIEEVEVKSVADFNHALEKYKGKP 457
SP|P0C0V0|DEGP_ECOLI   --EMSNKGKDQGVVVNNVKTGTPAAQIGLKKGDVIIGANQQAVKNIAELRKVLDSK---P 455
                         .*   ...  ***:*.:*: .:** * *:::*::*    ::  **.:*::.:.*:.     *

TR|G2J5T2|G2J5T2_HELPY KRFLVLDLNQGYRIILVK-- 475
SP|P0C0V0|DEGP_ECOLI   -SVLALNIQRGDSTIYLLMQ 474
                         .*.*::::*   * :
```

B

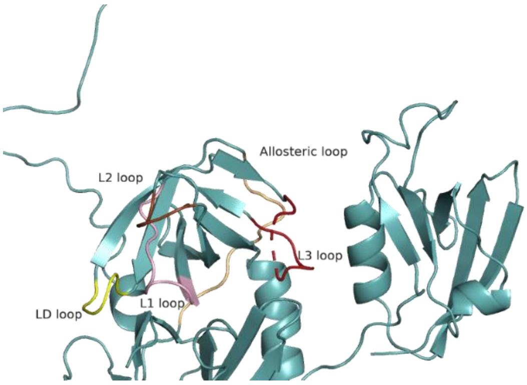

Figure S6. Sequence alignment of HpHtrA (G2J5T2) and *E. coli* DegP (P0C0V0). (A) HtrA contains a signal peptide (orange), protease domain (green) containing the catalytic triad (red), and the two PDZ domains (purple). The regulatory important loops LA (blue), LD, L1, L2, and L3 are highlighted in black boxes [2,3]. The putative allosteric ligand binding loop is shown in a red box. **(B)** The regulatory loops LD (yellow), L1 (rose-red), L2 (brown), L3 (red) and the allosteric loop (orange) were highlighted in the HpHtrA structure.

Figure S7

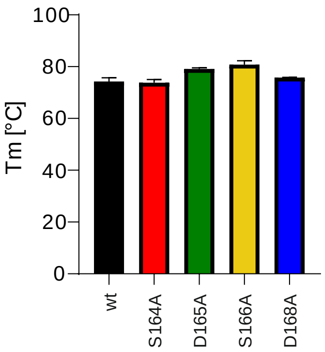

Figure S7. Melting temperatures of HpHtrA allosteric loop mutants. 4 µM recombinant HpHtrA wt, HpHtrA $S_{164}A$, HpHtrA $D_{165}A$, HpHtrA $S_{166}A$, and HpHtrA $D_{168}A$ were incubated with SYPRO Orange at a temperature ramp from 25°C-95°C (increase of 0.5°C per minute). Melting temperatures (Tm) [°C] ± S.D of HpHtrA wt, HpHtrA $S_{164}A$, HpHtrA $D_{165}A$, HpHtrA $S_{166}A$, and HpHtrA $D_{168}A$ are shown.

Figure S8

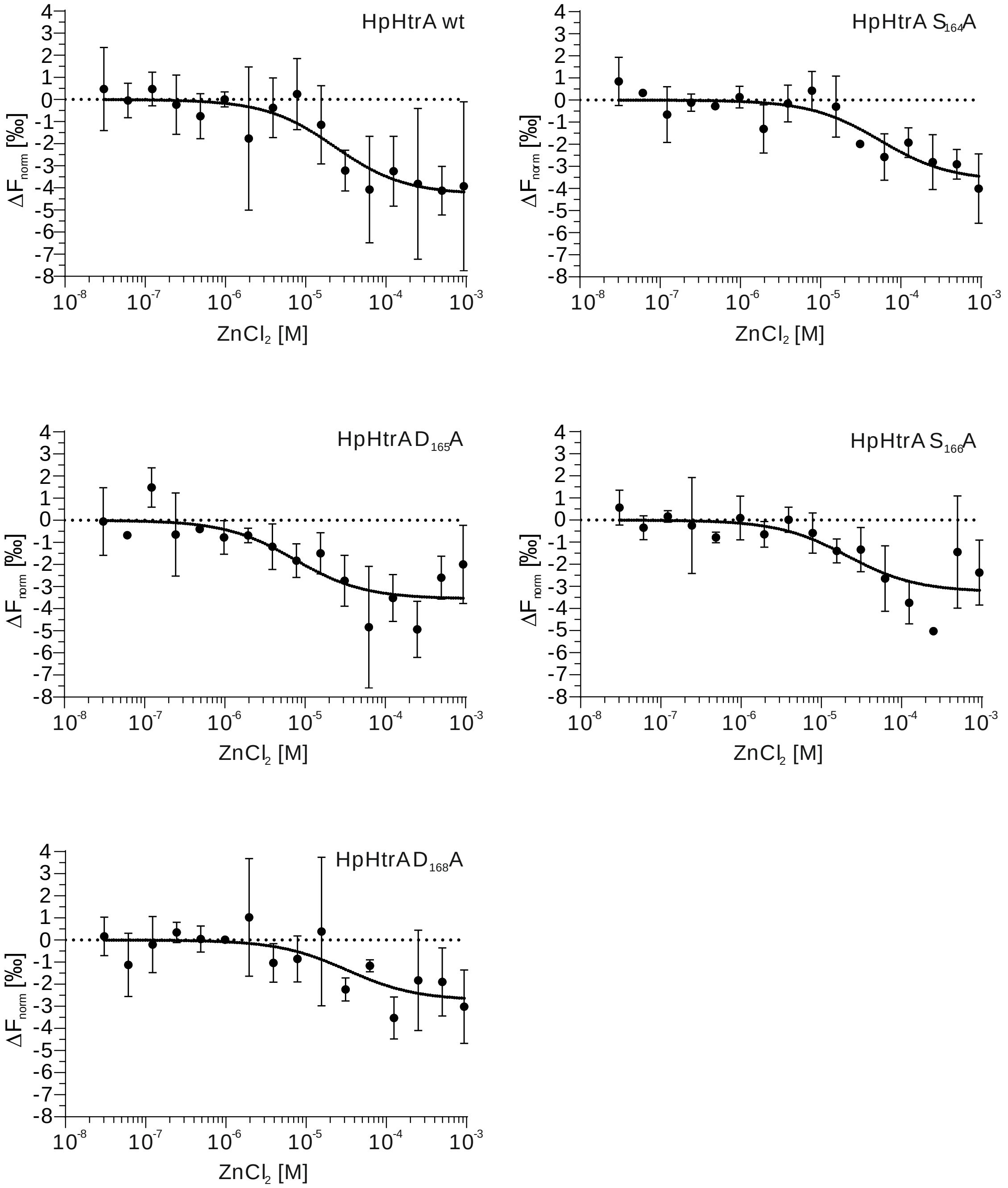

Figure S8. Dose response curves for the binding interaction between Zn^{++} and HpHtrA. HtrA wt, $S_{164}A$, $D_{165}A$, $S_{166}A$, and $D_{168}A$ were analyzed for binding to Zn^{++} by MST. 5 nM of HtrA proteins labelled with NT-650-NHS was kept constant, while $ZnCl_2$ was titrated from 1 mM - 30.5 nM. The difference in normalized fluorescence [‰] was plotted versus concentrations of $ZnCl_2$. Data represent three independent experiments.

Original gels and blots for Fig. 2

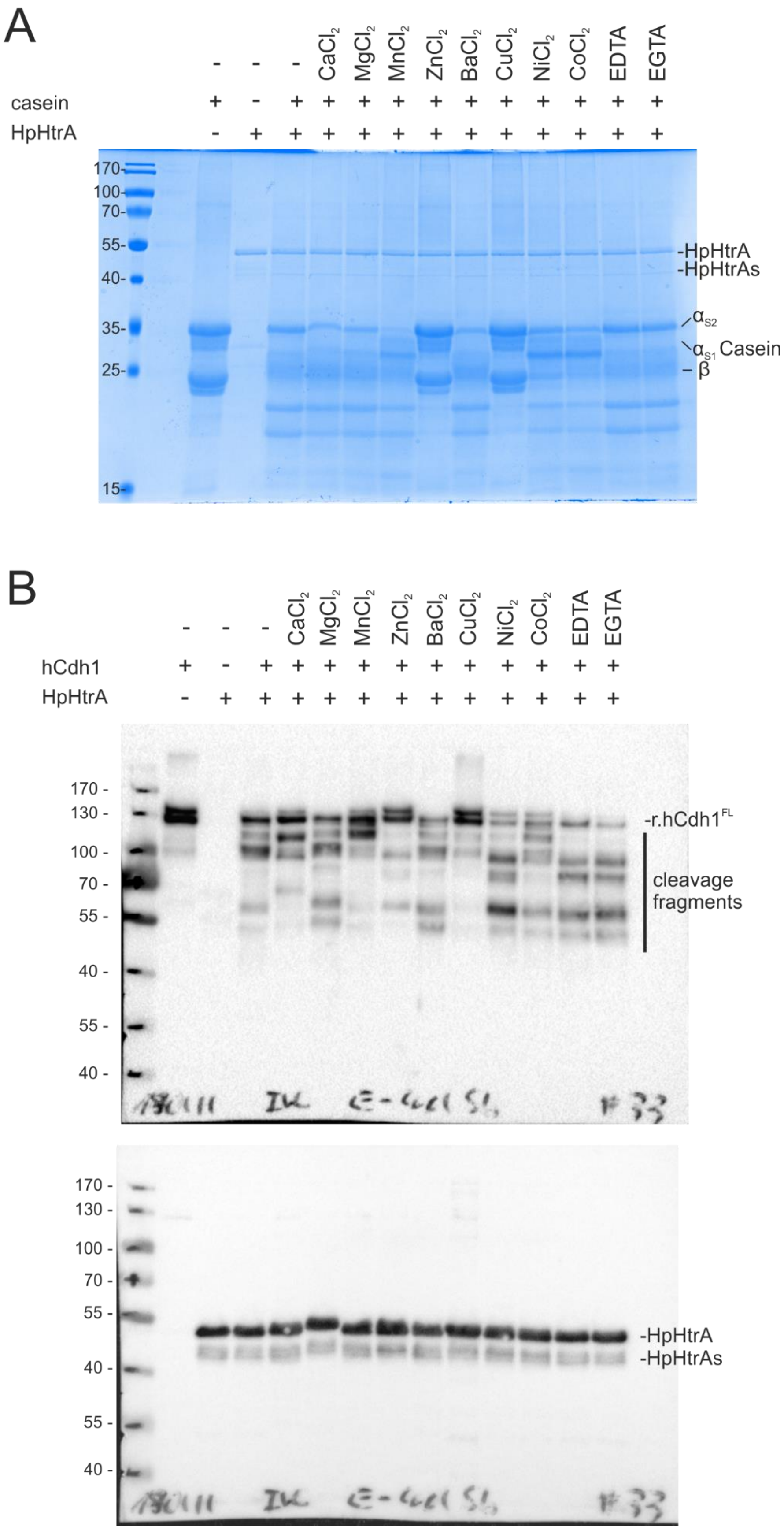

Original gels and blots for Fig. 3

B

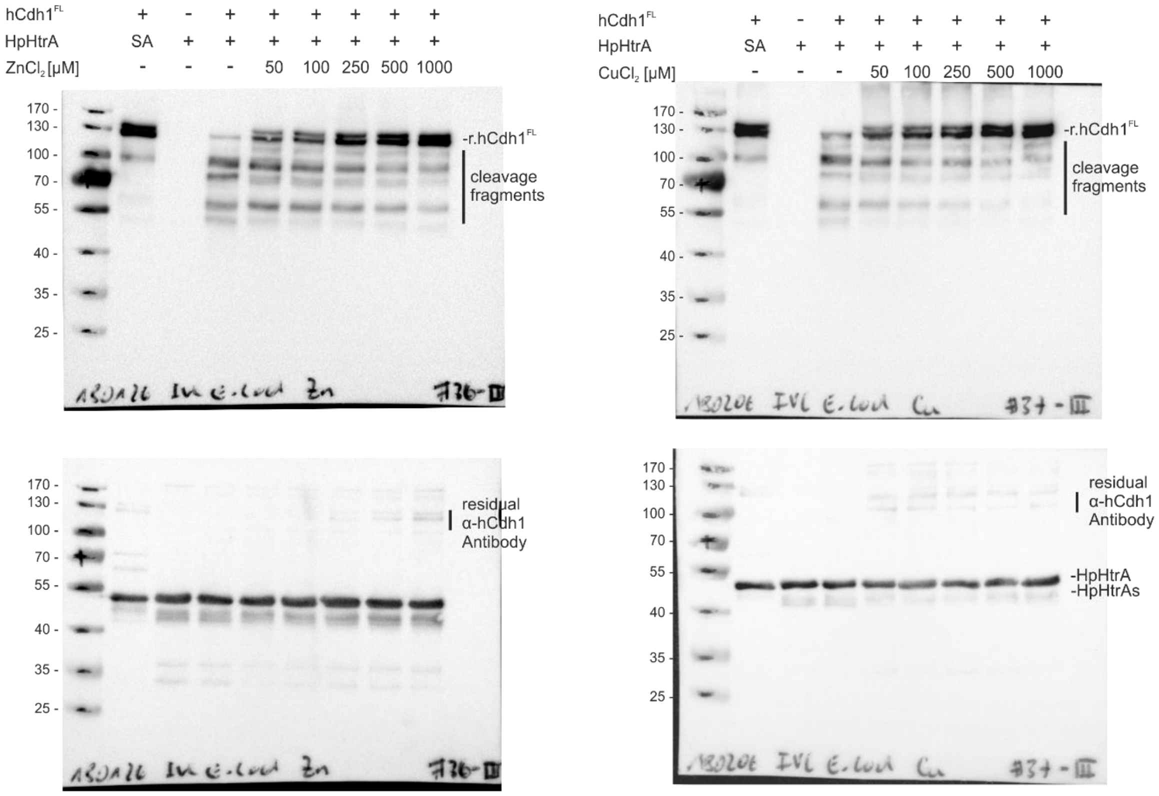

C

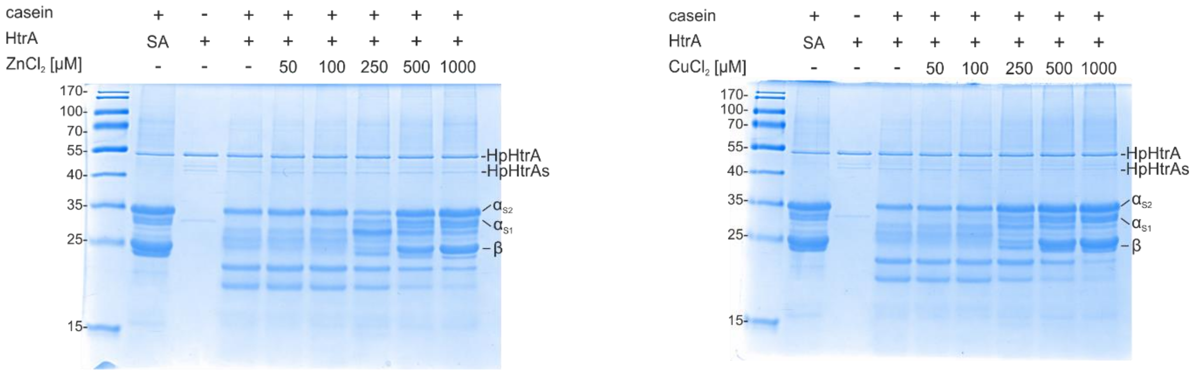

Original gels for Fig. 4

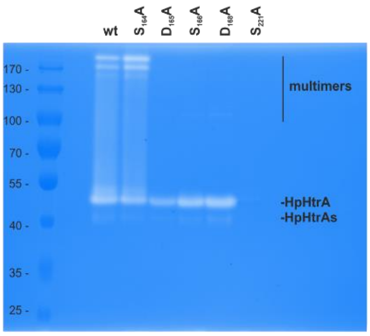

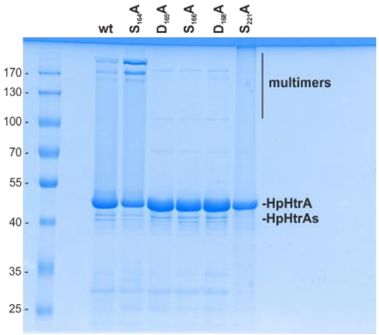

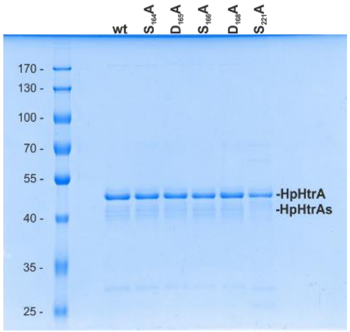

Original gels and blots for Fig. 5

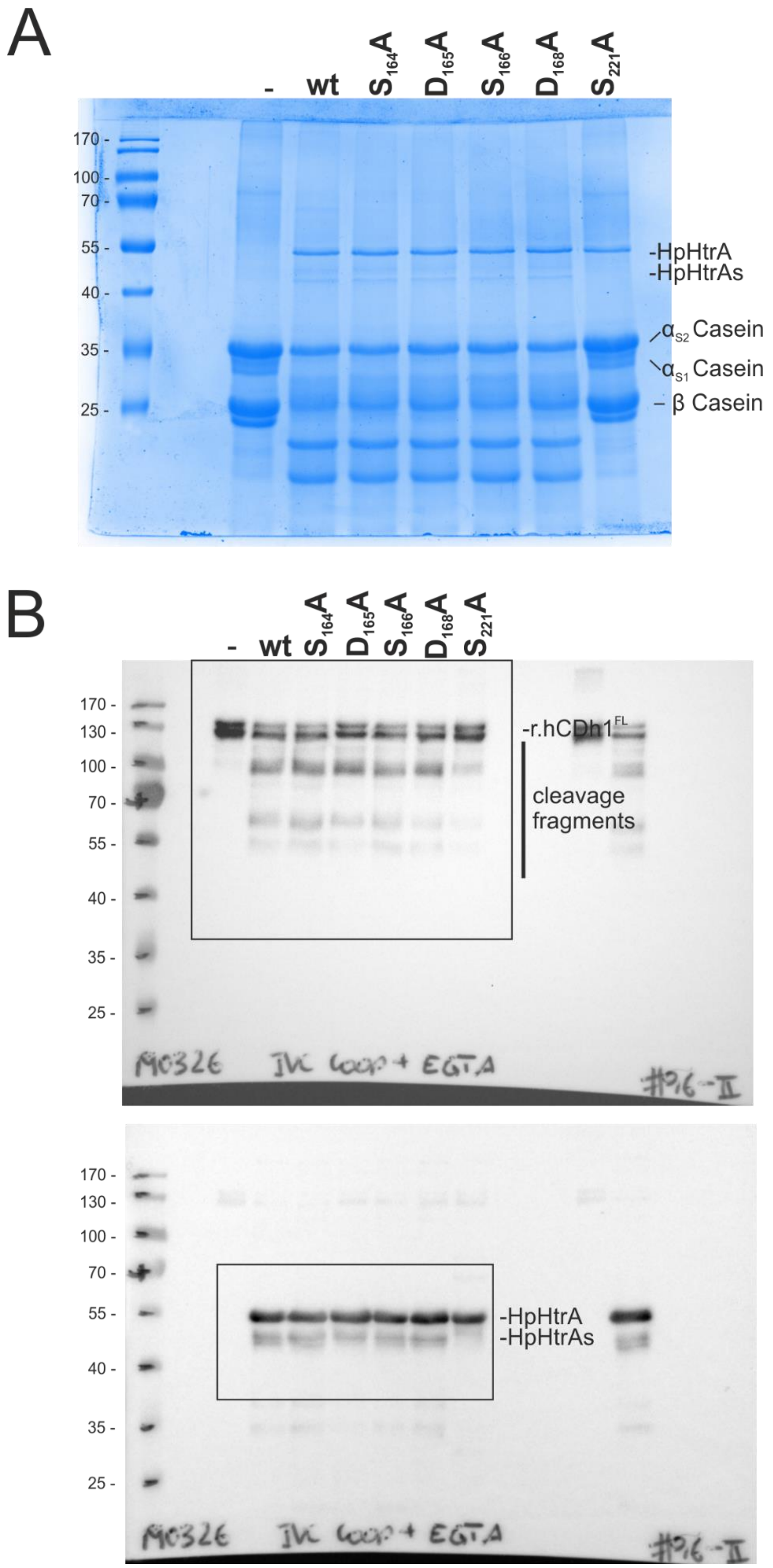

Original gels for Fig. 6

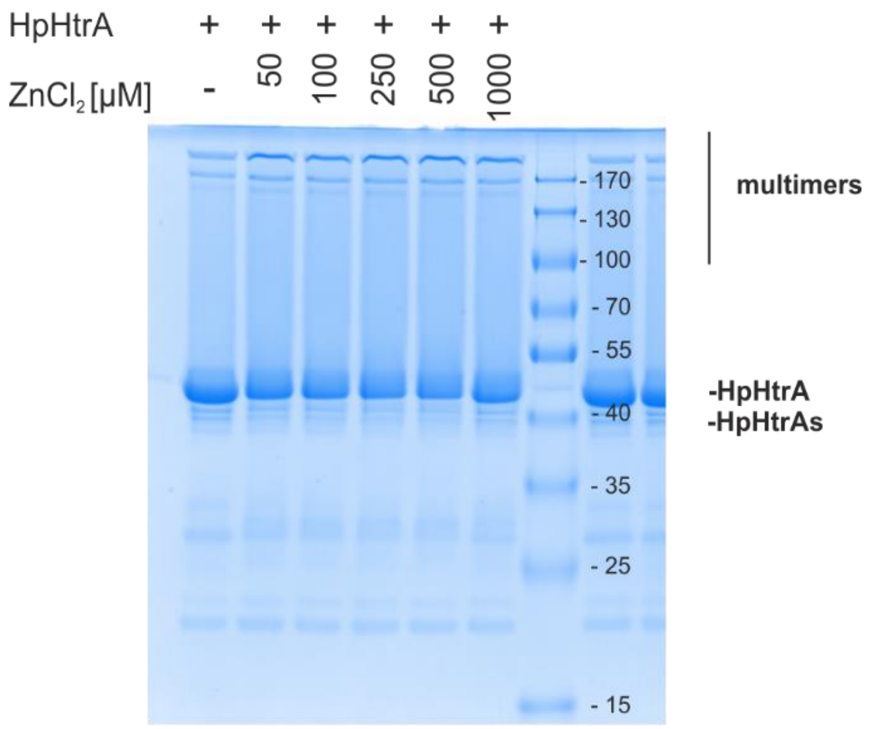

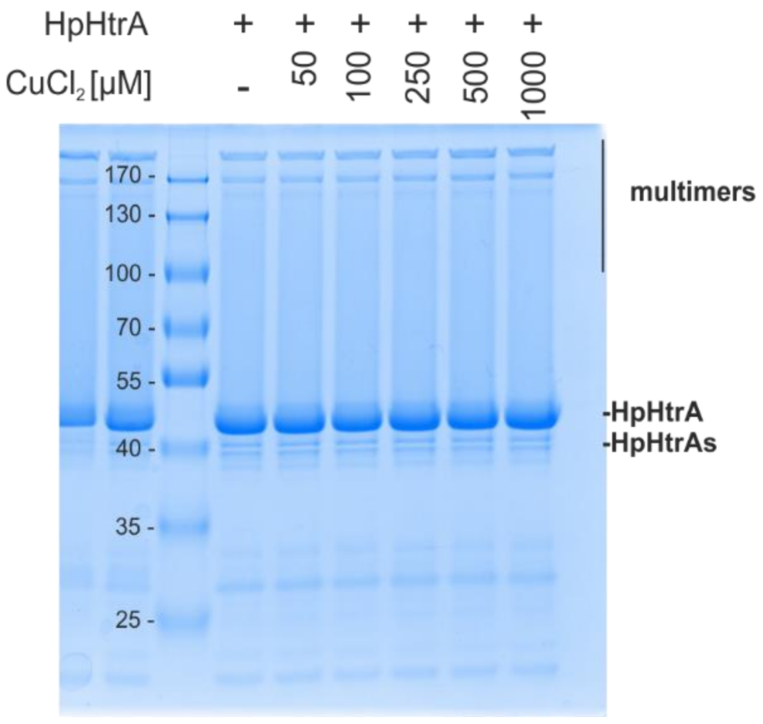

References

1 Tcherkasskaya, O. & Ptitsyn, O. B. Direct energy transfer to study the 3D structure of non-native proteins: AGH complex in molten globule state of apomyoglobin. *Protein Eng* **12**, 485-490, doi:10.1093/protein/12.6.485 (1999).

2 Marsh, J. W., Lott, W. B., Tyndall, J. D. & Huston, W. W. Proteolytic activation of Chlamydia trachomatis HTRA is mediated by PDZ1 domain interactions with protease domain loops L3 and LC and beta strand beta5. *Cell Mol Biol Lett* **18**, 522-537, doi:10.2478/s11658-013-0103-2 (2013).

3 Krojer, T. *et al.* Structural basis for the regulated protease and chaperone function of DegP. *Nature* **453**, 885-890, doi:10.1038/nature07004 (2008).
